# A randomized controlled experimental medicine study of ghrelin in value-based decision making

**DOI:** 10.1172/JCI168260

**Published:** 2023-06-15

**Authors:** Michal Pietrzak, Adam Yngve, J. Paul Hamilton, Robin Kämpe, Rebecca Boehme, Anna Asratian, Emelie Gauffin, Andreas Löfberg, Sarah Gustavson, Emil Persson, Andrea J. Capusan, Lorenzo Leggio, Irene Perini, Gustav Tinghög, Markus Heilig

**Affiliations:** 1 Center for Social and Affective Neuroscience, Department of Biomedical and Clinical Sciences, Linköping University, Linköping, Sweden.; 2 Department of Psychiatry, Linköping University Hospital, Linköping, Sweden.; 3 Center for Medical Imaging and Visualization and; 4 Division of Economics, Department of Management and Engineering, Linköping University, Linköping, Sweden.; 5 Section on Clinical Psychoneuroendocrinology and Neuropsychopharmacology, Translational Addiction Medicine Branch, National Institute on Drug Abuse Intramural Research Program and National Institute on Alcohol Abuse and Alcoholism, Division of Intramural Clinical and Biological Research, National Institutes of Health, Baltimore, Maryland, USA.; 6 National Center for Health Care Priority Setting, Department of Health Medicine and Caring Sciences, Linköping University, Linköping, Sweden.

**Keywords:** Metabolism, Addiction, Behavior, Neuroendocrine regulation

## Abstract

**BACKGROUND:**

The stomach-derived hormone ghrelin stimulates appetite, but the ghrelin receptor is also expressed in brain circuits involved in motivation and reward. We examined ghrelin effects on decision making beyond food or drug reward using monetary rewards.

**METHODS:**

Thirty participants (50% women and 50% men) underwent 2 fMRI scans while receiving i.v. ghrelin or saline in a randomized counterbalanced order.

**RESULTS:**

Striatal representations of reward anticipation were unaffected by ghrelin, while activity during anticipation of losses was attenuated. Temporal discounting rates of monetary reward were lower overall in the ghrelin condition, an effect driven by women. Discounting rates were inversely correlated with neural activity in a large cluster within the left parietal lobule that included the angular gyrus. Activity in an overlapping cluster was related to behavioral choices and was suppressed by ghrelin.

**CONCLUSION:**

This is, to our knowledge, the first human study to extend the understanding of ghrelin’s significance beyond the canonical feeding domain or in relation to addictive substances. Contrary to our hypothesis, we found that ghrelin did not affect sensitivity to monetary reward anticipation, but rather resulted in attenuated loss aversion and lower discounting rates for these rewards. Ghrelin may cause a motivational shift toward caloric reward rather than globally promoting the value of reward.

**TRIAL REGISTRATION:**

EudraCT 2018-004829-82.

**FUNDING:**

Swedish Research Council (2013-07434), Marcus and Marianne Wallenberg foundation (2014.0187) and National Institute on Drug Abuse/National Institute on Alcohol Abuse and Alcoholism Intramural Research Program.

## Graphical abstract

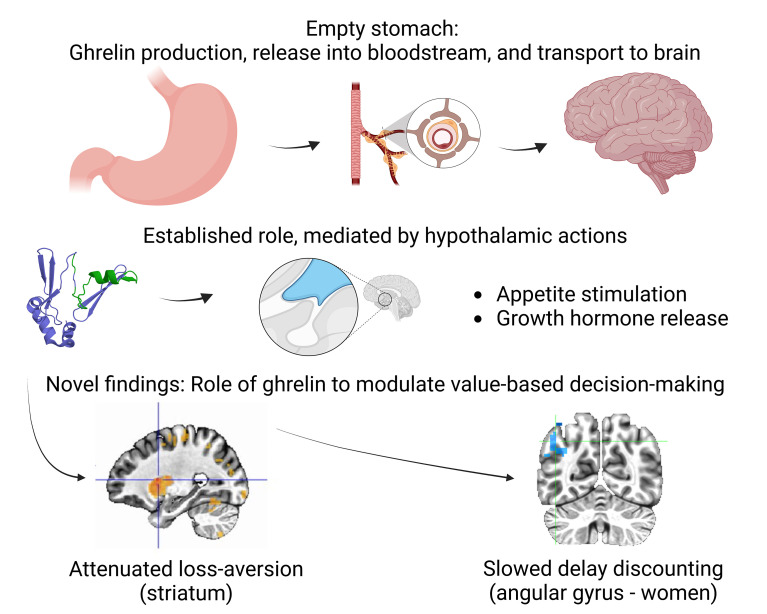

## Introduction

Ghrelin, a 28–amino acid hormone produced by endocrine cells of the stomach transmits a hunger signal to the brain that depends on distension of the stomach and relays information about nutrient availability. Actions of ghrelin are mediated by the ghrelin receptor (also referred to as the growth hormone secretagogue receptor [GHSR]), which is highly expressed in the hypothalamus, but also in extrahypothalamic brain areas (1). GHSR activation by ghrelin stimulates appetite by activating orexigenic and inhibiting anorectic neurons of the hypothalamus, and promotes growth hormone (GH) release from the pituitary.

Neurocircuitry mediating appetite regulation may also be a shared substrate of eating disorders and addiction (2). Accordingly, both animal and human studies indicate that ghrelin promotes motivation to seek alcohol (3) and may be involved in reward from other drugs (4). These findings may be related to GHSR expression in the ventral tegmental area (VTA), the origin of mesolimbic dopamine (DA) projections broadly involved in motivated behavior (5). It is, however, presently unknown whether ghrelin influences outcome valuation beyond food and addictive substances. Value-based decision making in humans has primarily been studied in the financial domain, but must rely on systems that precede monetary reward in evolutionary history (6). This suggests that appetite signals such as ghrelin may broadly influence decision making. Supporting this notion, a landmark study of judicial decision making found that the probability of a favorable ruling dropped just before a lunch break, and spiked back up after the meal (7). Although an alternative account of these findings has been proposed (8), a similar pattern of discontinuity around lunch has been shown for clinical decision making by surgeons (9). Prior findings in rats have suggested that ghrelin promotes impulsive decision making in pursuit of reward (10) and correlational data in humans have been consistent with this notion (11).

When administered i.v., ghrelin increases appetite in healthy volunteers (12) and promotes alcohol craving as well as self-administration in heavy drinkers (13, 14). It is unknown whether i.v. ghrelin influences decision making and its neural substrates beyond these domains. Here, we addressed this question using a double-blind, randomized placebo-controlled trial and an established infusion paradigm previously used to demonstrate the effects of ghrelin on appetite (12) (Supplemental Figure 1; supplemental material available online with this article; https://doi.org/10.1172/JCI168260DS1). We assessed ghrelin effects on neural representations of imminent reward and losses using the most widely used task that probes reward-related processing, the monetary incentive delay (MID) task (15–17) (Supplemental Figure 2A). In this task, anticipating rewards and losses elicits neural activations in overlapping areas within the striatum that scale with outcome magnitude. Neural activations in the MID task are thought to reflect striatal DA-release (18, 19). We then assessed ghrelin’s effects on delay discounting of monetary reward using a published fMRI-adapted task (20) (Supplemental Figure 2B). Because both rat and human data have suggested a relationship between ghrelin and impulsivity in pursuit of rewards (10, 11), we hypothesized that ghrelin would potentiate striatal activations to reward anticipation and result in steeper delay discounting.

## Results

### Plasma ghrelin and endocrine response

**Figure 1:**
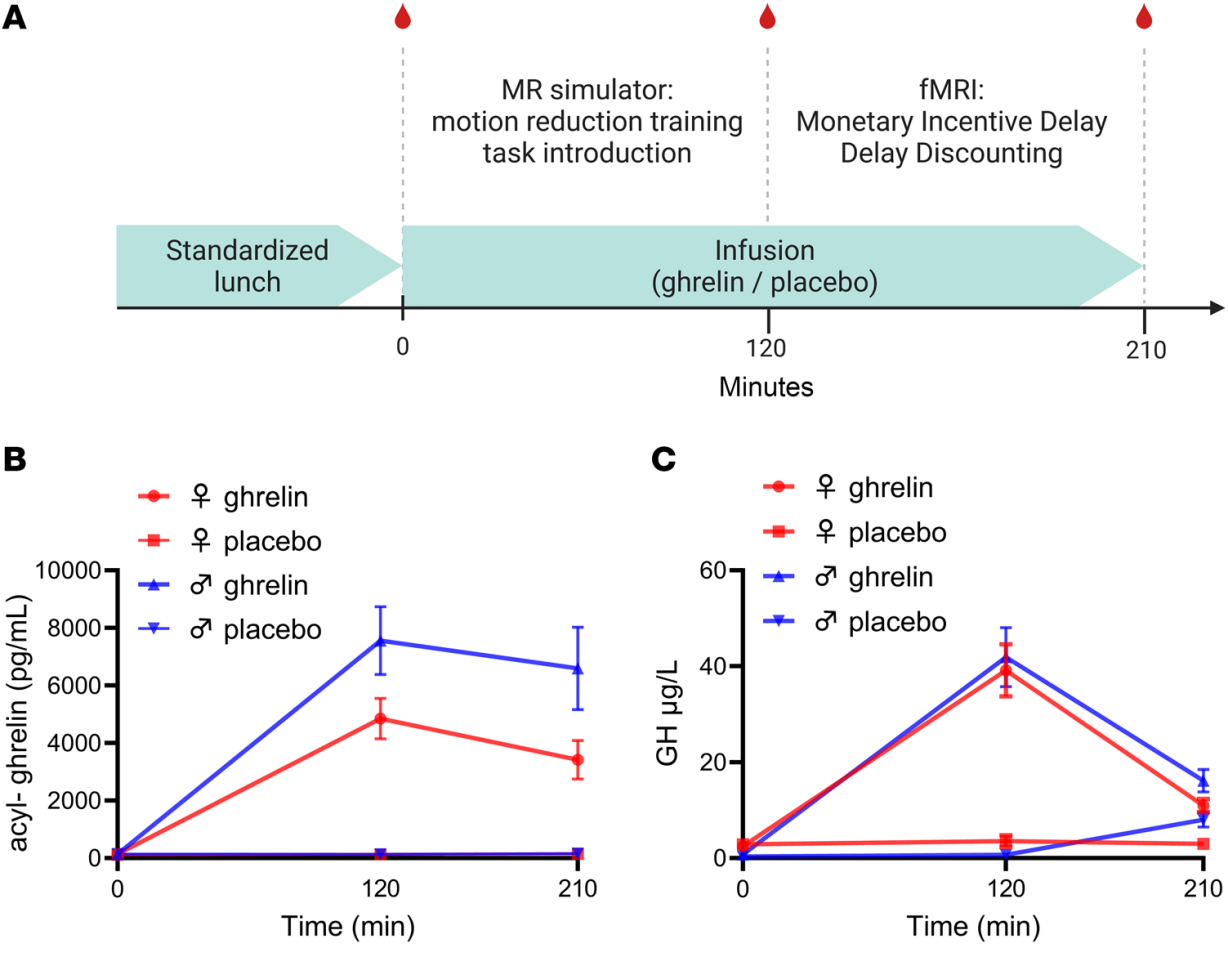
Procedures and manipulation checks. (**A**) Overview of study procedures. Red symbols represent blood draws. (**B**) Plasma levels of ghrelin (acyl-ghrelin) over the course of the 5 pmol/kg/min infusions. Levels achieved through the infusion were higher in male participants compared with female participants [Repeated measures ANOVA with sex as between-subject, and intervention and time as within-subject factors; *n* = 14 (men) or 13 (women). Main effect of sex: F(1,23)=5.17, *P* = 0.032; sex × intervention interaction: F(1,23)=5.14, *P* = 0.033. (**C**) Plasma levels of growth hormone in response to the ghrelin challenge were, however, virtually identical in men and women. Values are mean ± SEM.

In replication of prior findings (21), ghrelin infusions produced robust increases in circulating ghrelin (Figure 1B) and total ghrelin (Supplemental Figure 3). By using a standardized meal, we ensured that baseline, preinfusion levels of ghrelin were low in all participants on both sessions. Neither these basal ghrelin levels, nor those measured at any subsequent time point over the course of the infusion correlated with BMI or age. Ghrelin levels achieved through the infusion were somewhat higher in men than in women (Figure 1B). However, as a biomarker of bioactivity, we replicated the expected increase of plasma growth hormone (GH) after i.v. ghrelin and found it to be virtually identical in male and female participants, indicating equivalent bioactivity (Figure 1C). Adverse events, recorded and graded according to regulatory standards, were few, mild, and in no case assessed as having a likely or a possible relationship to study medication (Supplemental Table 7). These events were mostly related to the MR-scanning, and evenly distributed between ghrelin and placebo-sessions.

### MID task

#### Reaction times.

Reaction times in the task did not differ as a function of intervention (see Supplemental Results).

#### Outcome anticipation phase.

**Figure 2:**
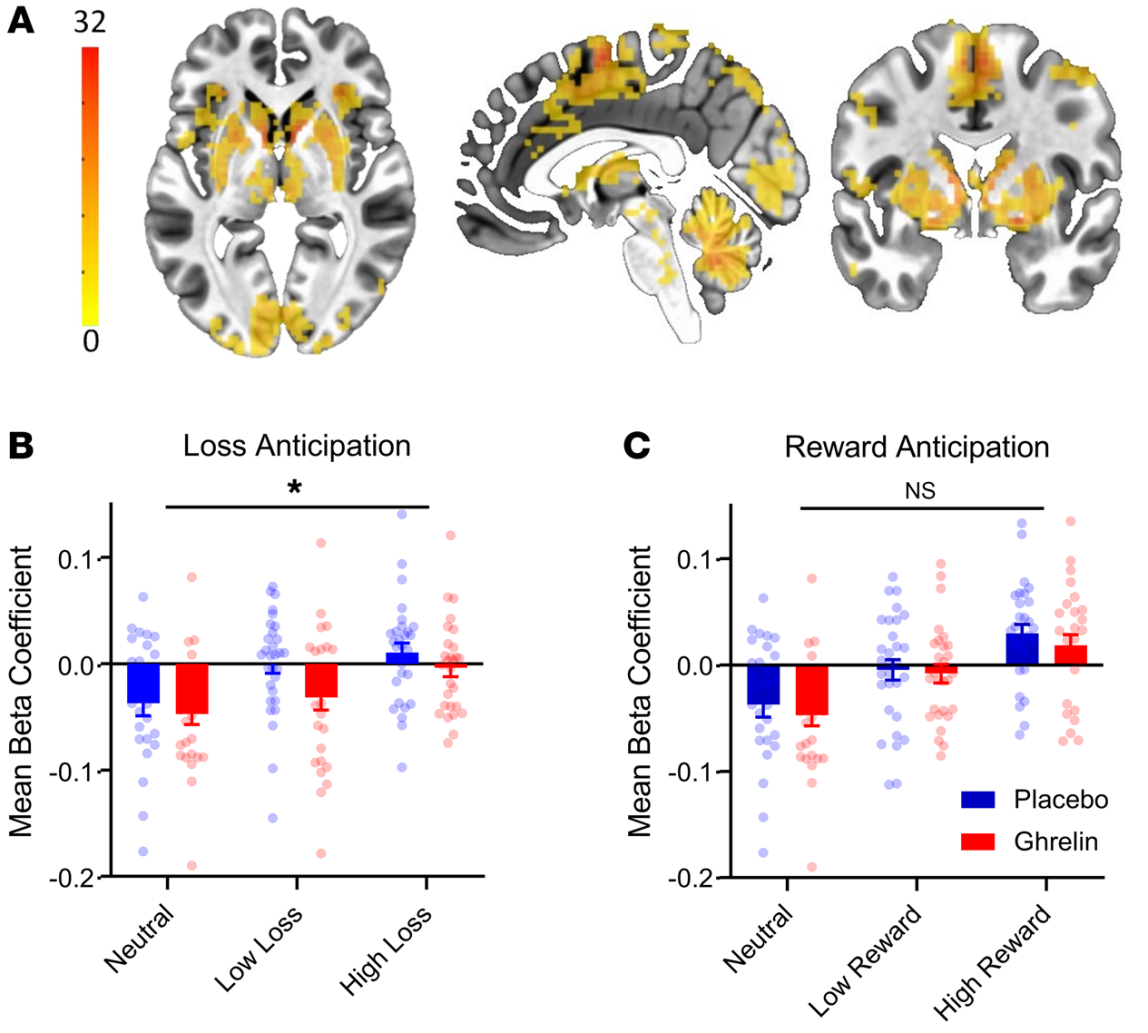
Results in the monetary incentive delay task. (**A**) Activation map of striatal cluster (1,734 voxels, MNI –2, 0, 1) identified by whole-brain analysis for main effect (*P* < 0.001) of anticipation (high loss – low loss – neutral – low reward – high reward). (**B** and **C**) Bar charts depicting extracted β-coefficients from the cluster shown in A. For loss anticipation (**B**), a 2 × 3 ANOVA (ghrelin/placebo × neutral/low loss/high loss) showed a main effect of anticipation (F[2,54]=17.1, *P* < 0.001, ηp^2^ =0.38), confirming that there was also a parametric increase in activity with loss magnitude. During loss anticipation, a main effect of Intervention was present (F[1,27]=5.88, *P* = 0.022, ηp^2^=0.18). For reward anticipation (**C**) a 2 × 3 ANOVA (intervention: placebo/ghrelin × anticipation: neutral/low reward/high reward) also found a main effect of anticipation (F[1,37]=36.9, *P* < 0.001, ηp^2^=0.58), confirming the parametric increase in reward-related activation. However, during reward anticipation, no effect of Intervention was found (*P* = 0.43). Bars are means + SEM; *n* = 28 / condition (placebo versus ghrelin). Colored panel represents F statistic. Asterisk denotes the main effect of intervention on loss anticipation; ns = nonsignificant effect of intervention on reward anticipation. Pairwise comparisons showed that brain responses to loss anticipation were attenuated by ghrelin (mean difference ± SEM: –0.018 ± 0.008, *P* = 0.022). Sex had no main (nor interaction) effect on either outcome anticipation.

Based on considerations presented in Methods, we carried out a single whole-brain Multi-Variate Modeling (3dMVM) analysis of outcome value (including all magnitudes of reward and losses) versus neutral outcome anticipation. This identified a large striatal cluster (1,734 voxels) associated with outcome anticipation (Figure 2A). A full list of brain regions significantly activated during outcome anticipation is in Supplemental Table 2. Activation locations for anticipation high reward > neutral and anticipation high loss > neutral are in Supplemental Tables 3 and 4.

During loss anticipation (Figure 2B), activation within the striatal cluster scaled with the magnitude of expected losses. There was also a main effect of intervention (*P* = 0.022, ηp^2^ = 0.18), and pairwise comparisons showed that brain responses to loss anticipation were attenuated by ghrelin (mean difference ± SEM: 0.018 ± 0.008, *P* = 0.022). During reward anticipation, activation within the striatal cluster also scaled with the magnitude of anticipated reward, but did so similarly under placebo and ghrelin conditions (Figure 2C). There was a main effect of anticipation (*P* < 0.001, ηp^2^ = 0.58; for detailed statistics, see figure legend), confirming the parametric increase in reward activation; but no effect of intervention (*P* = 0.426). Thus, ghrelin did not significantly alter neural activity in the striatum associated with anticipation of imminent reward, but attenuated activation during loss anticipation.

#### Feedback phase.

Whole-brain 3dMVM analysis of the feedback phase showed a main effect of reward feedback, with a large striatal cluster located in the left caudate nucleus compared with neutral feedback (± 0 SEK; 199 voxels; Supplemental Figure 9A). A full list of activations for feedback reward > neutral is in Supplemental Table 5. The whole-brain analysis also found a significant intervention × feedback interaction, with stronger activation of the posterior middle cingulate cortex (MCC) in response to reward during ghrelin compared to placebo (24 voxels; Supplemental Figure 9B).

#### Additional effects.

The main effects of ghrelin where observed outside the striatum during reward anticipation in the right calcarine gyrus (Supplemental Figure 10) and during loss anticipation in the right calcarine gyrus and left postcentral gyrus (Supplemental Figure 11). These were not pursued further.

### Delay discounting task

#### Behavioral results.

Reaction times in the task did not differ as a function of intervention (see Supplemental Results). Six subjects (4 women and 2 men) were excluded from final analysis; 4 due to erratic data (inconsistent to a degree that did not allow the discounting constant to be estimated), and 2 because their estimated *k* values were more than 2 SD from the overall mean, and approximately 10-fold higher than the group mean, both on placebo and ghrelin sessions.

**Figure 3:**
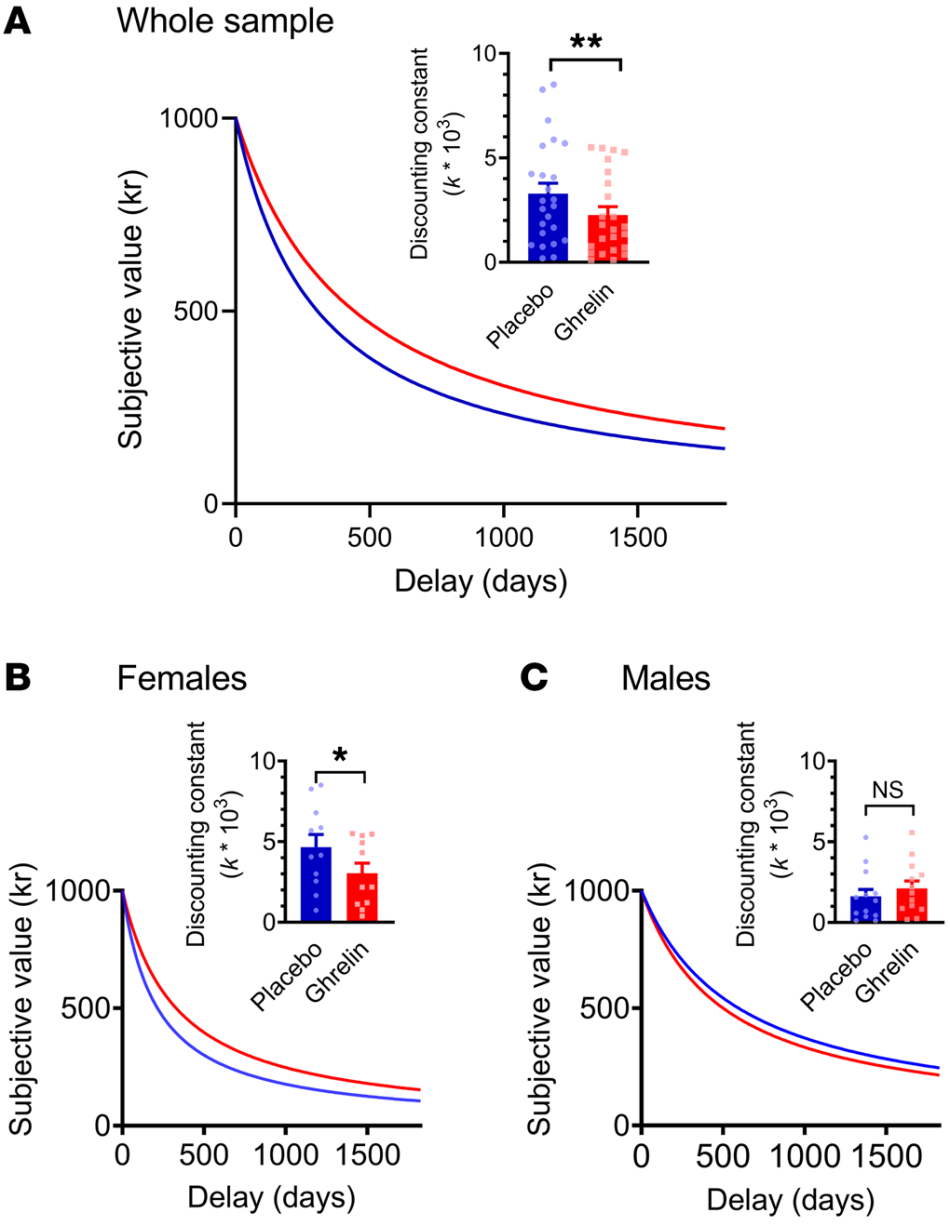
Behavioral results in the delay discounting task. *n* = 23 (12 men and 11 women). (**A**) Main graph: fitted hyperbolic discounting functions under placebo (blue) and ghrelin (red) conditions; Inset: mean ± SEM and individual discounting constant *k* under the respective condition. (**B**) discounting constant for women only; (**C**) discounting constant for men only. There was a main effect of intervention (F[1,22]=11.8, *P* = 0.002, ηp^2^=0.35), a main effect of sex (F[1,22]=7.1, *P* = 0.014, ηp^2^=0.24), and a trend for an intervention × sex interaction (F[1,22]=3.2, *P* = 0.09, ηp^2^=0.13). Post-hoc Newman-Keuls tests indicated that, under the placebo condition, female participants had a steeper discounting (higher *k* value) than male participantss (*P* = 0.01); and that the main effect of treatment was driven by women (ghrelin versus placebo: female participants - *P* = 0.001; male participants – *P* = 0.26).

In the final analysis (Figure 3), there was a main effect of intervention (*P* = 0.002, ηp^2^ = 0.35), a main effect of sex (*P* = 0.014, ηp^2^ = 0.24), and a trend for an intervention × sex interaction (*P* = 0.09, ηp^2^ = 0.13). Post hoc Newman-Keuls tests indicated that, under the placebo condition, female participants had a steeper discounting (higher *k* value) than male participants (*P* = 0.01); and that the main effect of intervention was driven by female participants (ghrelin versus placebo: female participants *P* = 0.001; male participants *P* = 0.26).

#### fMRI results.

**Figure 4:**
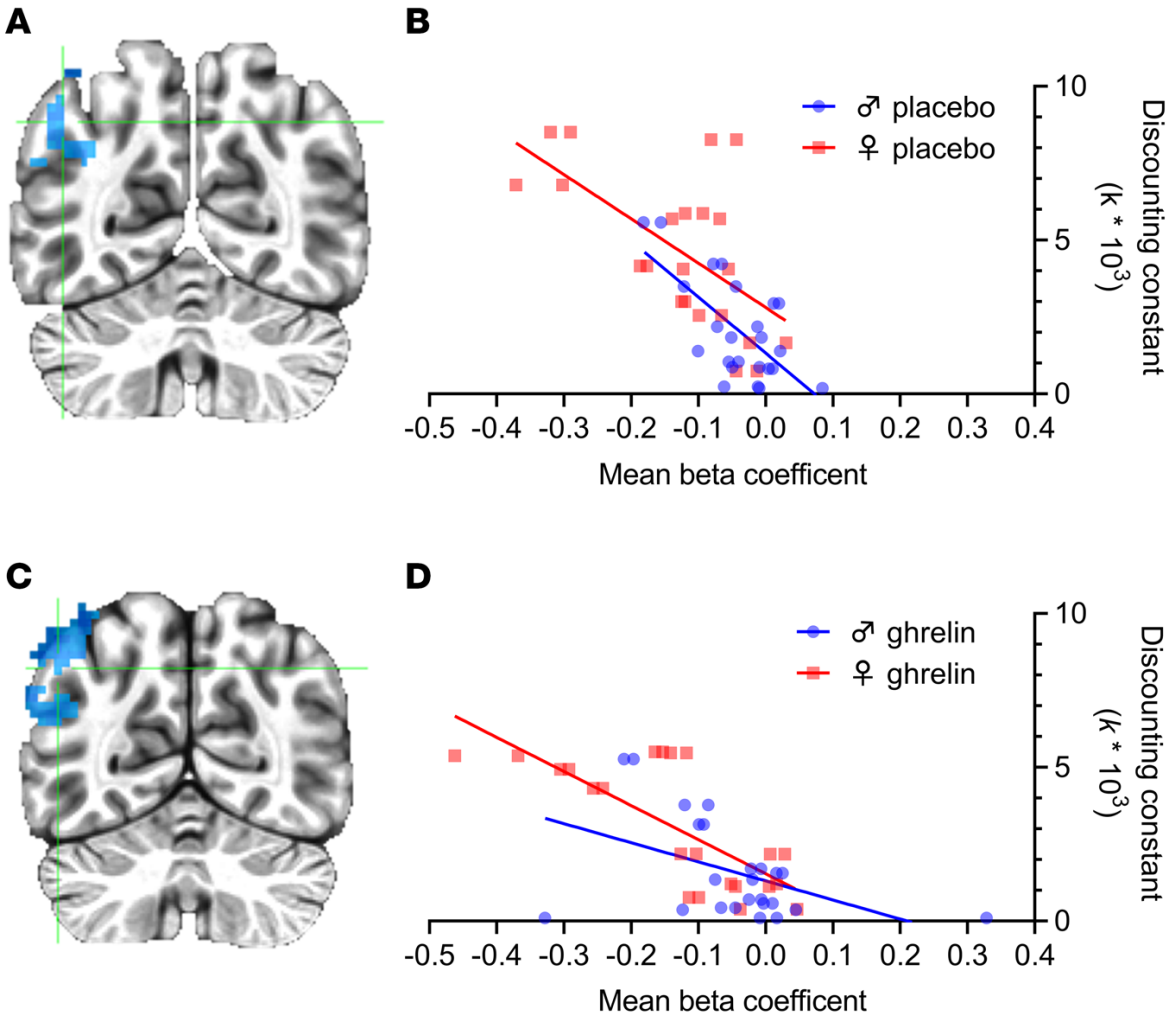
Whole-brain analysis of the relationship between behavior and neural activations in the delay discounting task. Under the respective condition, analysis used 2 data points/subject, obtained from less-now and more-later choices, respectively (*n* = 23; 12 men and 11 women). (**A**) Parietal cluster (MNI coordinates: –45, –63, 43; cluster size: 199) identified as main effect of *k* value under placebo conditions; (**B**) Correlation between neural activity (β-coefficients) and the *k* value under placebo conditions: Slope –0.02, R^2^=0.55, *P* = 0.00005; (**C**) Overlapping parietal cluster (MNI coordinates: –49, –64, 42; cluster size: 294) identified by the same analysis as in panel **A**, but under ghrelin conditions; (**D**) Correlation between neural activity (β-coefficients) and *k* value under ghrelin conditions: Slope: –0.012; R^2^=0.53, *P* = 0.001. Bootstrapping test with 10,000 permutations found that the 95% confidence interval for the difference between slopes, 0.0084, was 0.0010–0.0177 and thus significantly different from 0. The correlation results were very similar when women and men were analyzed separately.

Because the *k* value summarizes the rate of temporal discounting for an individual, we searched for the neural substrates of this process by carrying out a whole-brain analysis in search of neural activity associated with *k* value. Both under placebo (Figure 4A) and ghrelin (Figure 4C) conditions, the main effect of *k* identified overlapping clusters in a region of left posterior parietal cortex that included the angular gyrus (Figure 4, A and C; see Supplemental Figure 12 for a conjunction analysis demonstrating the overlap). A negative correlation between neural activations within these clusters and *k* value was significant under both conditions, a result that remained when correlations were examined separately for men and women (placebo: R^2^ = 0.55, *P* = 0.00005, Figure 4B; ghrelin: R^2^ = 0.55, *P* = 0.00005, Figure 4D). The slopes of the correlation between *k* value and β-coefficients were significantly different under placebo and ghrelin conditions (*P* < 0.05; see Figure 4), suggesting that ghrelin influenced neural processing associated with temporal discounting.

**Figure 5:**
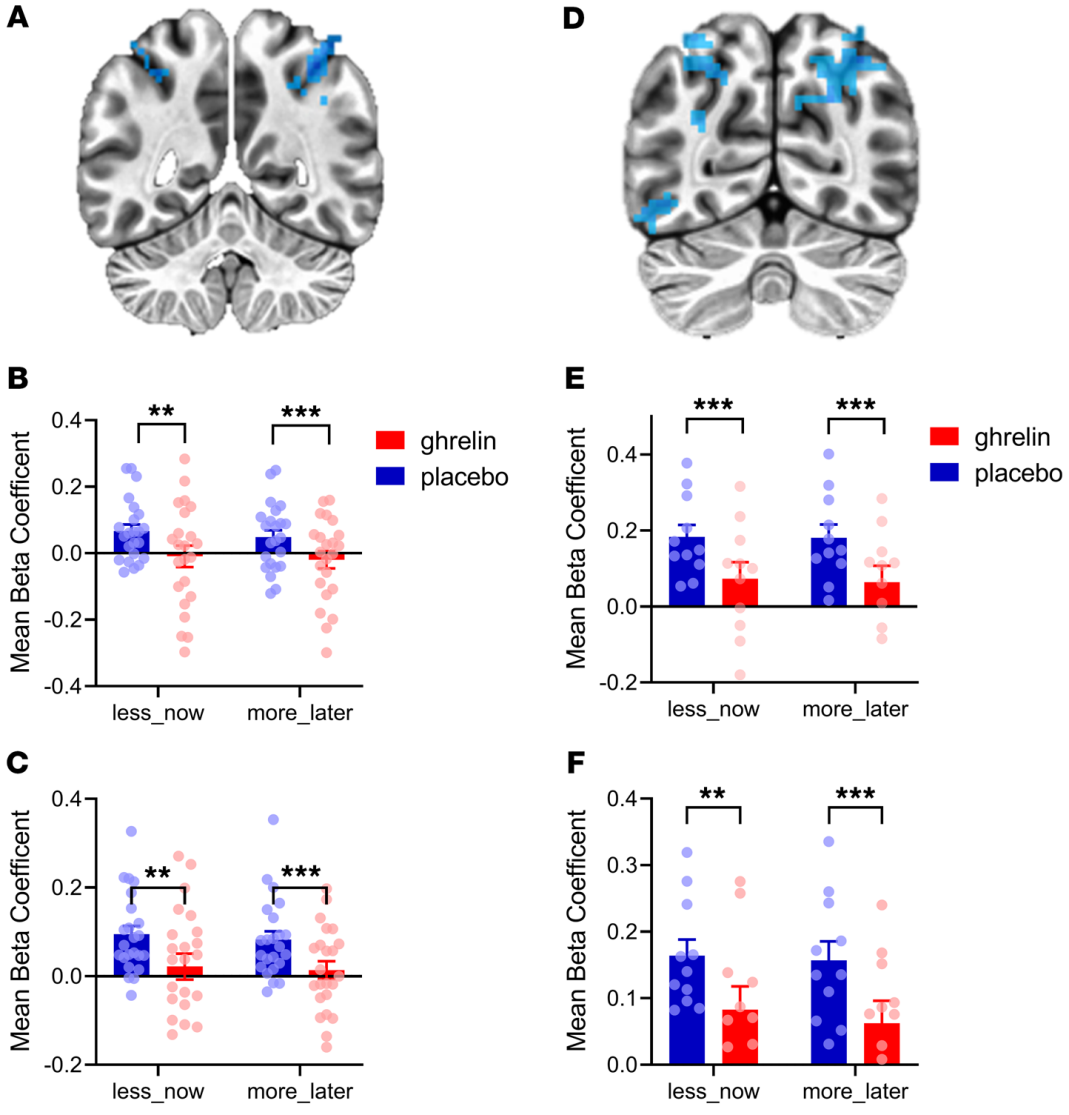
Whole-brain analysis for main effect of intervention in the delay discounting task. *n* = 23 (12 men and 11 women). (**A**) Bilateral cluster of deactivation (MNI coordinates: 35, –60, 48; cluster size: 248; MNI coordinates: –43, –48, 56; Cluster size: 179) Analysis of β-coefficients extracted from these clusters showed that, both for less-now and more-later choices, ghrelin produced a deactivation both in the left hemisphere (**B**) less-now choices (F[1,22]=11.7, **P = 0.002, ηp^2^=0.35) and more-later choices (F[1,22]=25.6, ***P < 0.001, ηp^2^=0.54) and right hemisphere (**C**) less-now choices (F[1,22]=13.7, **P = 0.001, ηp^2^=0.38) and more-later choices (F[1,22]=17.9, ***P < 0.001, ηp^2^=0.45). When analyses were stratified by sex, similar results were replicated in female participants (**D**–**F**), (**E**) less-now choices (F[1,10]=23.6, ***P < 0.001, ηp^2^=0.70) and more-later choices (F[1,10]=31.4,*** *P* < 0.001, ηp^2^=0.76), (**F**) less-now choices (F[1,10]=17.8, **P = 0.002, η^2^=0.64) and more-later choices (F[1,10]=28.1, ***P < 0.001, ηp^2^=0.74), while no significant whole-brain effect was found in male participants. Bars are means ± SEM. (**D**) MNI coordinates: –33, –68, 43; 31, –65, 44 and –44, –57, –15; Cluster sizes are 408, 288, and 177, respectively.

In a separate whole-brain analysis, we found significant main effects of both choice and intervention. The main effect of intervention identified bilateral clusters in the posterior parietal lobe that in the left hemisphere overlapped with that identified in Figure 4. Analysis of β-coefficients extracted from these clusters showed that, both for less-now and more-later choices, ghrelin produced a deactivation both in the left and the right hemisphere (*P* < 0.002 or 0.001, respectively; for detailed statistical results, see Figure 5). The effect of choice also identified areas with increased activity during less-now versus more-later decisions in the left precuneus and bilateral calcarine gyri within primary visual cortex. We also saw this effect in dorsal anterior cingulate cortex (dACC) extending to medial prefrontal cortex (Supplemental Figure 13). In this cluster, reduced activity for both more-later and less-now decisions was observed during ghrelin versus placebo sessions. We also found a main effect of sex in a region of the left angular gyrus that overlapped with the findings seen separately in the *k* value, choice, and intervention analyses. In this region, women showed more deactivation than men across levels of choice and intervention factors.

Because the behavioral effects of ghrelin on delay discounting were driven by women, we also carried out analyses of choice and intervention effects stratified by sex, although these have to be considered exploratory, as they were not preplanned, and the study was not a priori powered to detect sex-dependent effects. The effects identified in the full sample were replicated in female participants. Importantly, an additional main effect of choice was observed in women in the same bilateral posterior parietal cluster for which there was an effect of intervention in the full-sample analysis (Supplemental Table 6 and Supplemental Figure 14). Specifically, we saw more deactivation for more-later versus less-now decisions in this region. This finding had considerable overlap with the left parietal region showing a main effect of *k* value across sessions in the full sample analysis. In contrast to the female participants, and in agreement with the behavioral findings, no significant effects were found in male participants.

## Discussion

We examined putative neural representations of imminent monetary rewards and losses using an established probe, the MID task (15), and were able to robustly replicate meta-analytic findings of a large striatal region activated by both rewards and losses (17). Neural activations within this region scaled with the magnitude of anticipated rewards as originally described (15), and were unaffected by ghrelin. Activations in this region also scaled with the magnitude of anticipated losses under placebo conditions, but these activations were significantly attenuated by ghrelin. We then examined the effects of ghrelin on delay discounting of monetary rewards and found that discounting was less steep under ghrelin infusion versus placebo, an effect driven by female participants. The fMRI analysis identified a region within left posterior parietal cortex where neural activation, irrespective of biological sex, was strongly and negatively correlated with *k*, the parameter that summarizes discounting rates. This region included the angular gyrus, a multimodal hub involved *i.a*., in numerical processing and construction of temporal context (22–24). A separate analysis found that ghrelin attenuated neural activations in an overlapping posterior parietal region, both during choices that favored less-now over more-later outcomes, and during those that reflected the opposite preference. Like the behavioral findings, this effect was driven by female participants. Overall, several control analyses indicated that effects of ghrelin seemed to be specific and unlikely to be driven by adverse effects, generally impaired performance, or impaired task engagement.

Our findings stand in contrast to our a priori hypotheses. Peripheral ghrelin has been proposed to promote appetite and increase the reward value of food in part by potentiating mesolimbic DA signaling (25–28). It is widely thought that a key role of the DA system is to generate motivational signals that promote efforts to obtain reward (6). Mesolimbic DA signaling presumably evolved to promote motivation for fundamental rewards such as food, but in present-day humans, it appears to encode motivational signals that generalize beyond those basic physiological needs. For instance, DA signaling is critical for acute reinforcement from addictive drugs (29). Accordingly, effects of ghrelin on DA signaling have been suggested to reflect shared biological mechanisms of obesity and addiction (2), and ghrelin promotes motivation to obtain alcohol in both mice and humans (3, 5, 13, 14). Effects of ghrelin on food reinforcement are also significantly reduced after dopamine depletion in the VTA following local administration of the dopaminergic neurotoxin 6-hydroxydopamine (30).

Neural activations in the MID task as assessed with fMRI are highly correlated with, and likely reflect, striatal DA release (18, 19). Our hypothesis was therefore that striatal activations to reward anticipation in the MID task would be potentiated by ghrelin. This hypothesis was not supported. Instead, we found evidence that ghrelin reduces sensitivity to loss. This finding is consistent with a prior report in which endogenous ghrelin levels correlated with continued gambling despite losses (31). Reduced sensitivity to losses and punishment has been reported in populations with elevated impulsivity, such as people with addictive disorders (32) and could be a mechanism through which ghrelin promotes drug seeking and taking.

Effects of ghrelin on delay discounting were equally unexpected. Our simplistic a priori hypothesis was that increased motivation for food would generalize across reward categories and make people prioritize immediate rewards over those that are distant in time. Findings in rodents, obtained using food rewards, supported this notion (10). In contrast, using monetary rewards in humans, we found just the opposite, in line with correlational data from underweight patients with anorexia nervosa, in whom high desacyl-ghrelin concentrations correlated with an increased preference for delayed monetary rewards (33). Our delay discounting findings were consistent. First, both behavioral and neural effects of ghrelin were driven by effects in women. Second, the neural effect mapped to an area in the posterior parietal cortex that on the left side overlapped with that independently identified by an analysis of *k* value associated activations, irrespective of intervention. An inverse correlation of neural activation in this area and delay discounting rates is consistent with previously reported findings (34, 35) and has been implied in cognitive control required to forego immediate rewards in favor of those that are distant in time. This area includes the angular gyrus, a heteromodal region that displays marked lateralization (22).

Meta-analyses have linked angular gyrus activity, primarily in the left hemisphere, with number processing and conflict resolution, in particular in go/no-go–type tasks (22, 36). This role is consistent with a recently proposed conceptual framework, according to which the angular gyrus enables online dynamic buffering of representations that span multiple sensory domains and are extended in time (23). Decisions that involve intertemporal choices between monetary rewards — which, in turn, can represent multiple types of tangible rewards — clearly fit this conceptualization. Furthermore, lesions to the dominant parietal lobe that affect the angular gyrus result in Gerstmann syndrome, a key symptom of which is acalculia (37). Effects of ghrelin on the angular gyrus would presumably have to be indirect, since this area is not known to express GHSR (1). The effects of ghrelin to decrease rates of temporal discounting and attenuate loss sensitivity in women might be related, since discounting of temporally distant rewards can be viewed as valuation of the risk that they will be lost (38). It can be speculated that decreased aversion to monetary losses and less steep discounting of monetary rewards in response to increased ghrelin levels reflects a shift in which a hunger signal provided by ghrelin causes the individual to prioritize food reward over other reward categories. Under conditions when energy stores need to be replenished, activity of ghrelin to selectively promote motivation for food, at the expense of other rewards, would clearly be more adaptive than a nonselective potentiation of reward values. Because we failed to anticipate this distinction, we did not collect hunger ratings, and this hypothesis will therefore have to await testing in future studies.

Finally, it is unclear why effects of ghrelin in the delay discounting task appear to be restricted to women, while attenuated loss aversion affected men and women alike. This apparent discrepancy is unlikely to be related to pharmacokinetic differences, as ghrelin levels achieved through the infusion were somewhat higher in men than in women, and the biomarker of ghrelin activity, GH release, was virtually identical between male and female participants. A methodological issue may account for the observation that women drove the effect of ghrelin in the delay discounting task. In agreement with published findings (39), baseline discounting rates in women under placebo conditions were markedly higher than those in men and may therefore have been more sensitive to reduction by ghrelin. However, we also note that sex differences have been reported for the role of ghrelin in the control of energy balance and could reflect an interaction between ghrelin and sex hormones (40).

Among the strengths of our study is its randomized, placebo-controlled, double-blind design and biomarker confirmation of GHSR activation. This allowed us to establish a causal role of ghrelin in humans for cognitive processes outside appetite regulation and food- or drug-related cues. Our sample size was based on an a priori power analysis and allowed us to robustly replicate several previously published findings in the MID task (17). To ensure generalizability, we balanced inclusion of men and women. However, since we did not have a priori hypotheses regarding sex-specific effects, the study was not powered to specifically analyze ghrelin × sex interactions. Our exploratory analyses suggested that behavioral and neural effects of ghrelin on delay discounting may have been selective for women, but these findings, based on limited sample sizes, are preliminary.

In summary, our results support the general notion that ghrelin plays a role in processes subserving value-based decision making beyond its established role as an appetite-stimulating hormone. They do not, however, support predictions based on animal studies that ghrelin generally enhances the value of prospective rewards through actions on mesolimbic DA signaling.

## Methods

### Overview

An overview is shown in Figure 1A, and details are provided in Supplemental Table 1. This was a within-subject, cross-over, double-blind, randomized placebo-controlled trial consisting of 3 visits. Visit 1 was for screening and eligibility assessment. On visits 2 and 3, participants received i.v. ghrelin or placebo, respectively, in a within-subject, randomized, counterbalanced design, as previously described (13, 14, 21).

### Participants

Participants (*n* = 30, 15 women and 15 men) were healthy volunteers recruited using ads and fliers. Detailed eligibility criteria are provided in the Supplemental Materials. In brief, participants had to be 18–65 years old (mean ± SEM: 26 ± 1.44; men: 25.27 ± 1.11; women: 26.67 ± 3.02; ns), and in good health as determined by medical history, physical exam, electrocardiogram, and lab tests. Female participants had to provide a negative urine pregnancy test before each study session. Participants were normal weight, with BMI (mean ± SEM: 23.95 ± 0.51; men: 24.22 ± 0.5; women: 23.77 ± 0.82; ns). A CONSORT diagram showing disposition of participants is in Supplemental Figure 1, and an overview of study visits in Supplemental Table 1. Additional participant details are provided in Supplemental Table 8.

### Visit timeline

Following inclusion, written informed consent, and randomization, participants attended 2 visits, between 1 and 6 weeks apart. During these visits, they received a standardized meal, followed by i.v. ghrelin or placebo in counterbalanced order while they underwent MRI. Both MRI visits followed the same sequences (Figure 1A). Participants had 2 i.v. catheters inserted, 1 for ghrelin/placebo infusion and the other for collection of blood samples. They received a light standardized lunch and had a baseline blood sample (T1) collected for subsequent analysis of ghrelin levels and other biomarkers. The i.v. line was then connected to a portable, MR-compatible infusion pump. Next, participants moved to a separate room where they performed motion reduction training inside a (nonmagnetic) mock MRI simulator (Psychology Software Tools Inc.) to habituate to the scanner environment using a feedback system. Participants also performed practice versions of the tasks outside the simulator to familiarize themselves with the structure of the tasks and the response system. A second plasma sample was collected approximately 15 minutes before scanning (T2). The infusion was ongoing as participants entered the scanner and continued throughout the scan. A final blood sample was collected at the end of the scan (T3).

### Standardized meal

To minimize the influence of endogenous ghrelin levels, participants were provided with a standardized meal before sessions (beef patties, red wine sauce, and cooked potatoes; energy content 456.1 kcal; macronutrient content: carbohydrates, 47.6 g; total fat 16.7 g; saturated fat 5.5 g; polyunsaturated fat 1.9 g; monounsaturated fat 7.8 g; protein 25.4 g; fiber 4.6 g).

### Drug administration

Leiden University Medical Centre Research Pharmacy (Leiden, The Netherlands), delivered sterile, freeze-dried ghrelin powder (cGMP Human Acyl-Ghrelin) in individual 250 μg subject vials to the Hospital Pharmacy in Linköping. Each vial had study-specific labelling in Swedish. Infusion bags were prepared by the pharmacy as 200 mL of 0.9% NaCl (placebo) or 2.5 μg/mL ghrelin. The pharmacy dissolved each vial of 250 μg sterile, freeze-dried ghrelin powder in 5 mL of 0.9% NaCl, and then added the content of 2 vials (10 mL, 500 μg) to an infusion bag with 0.9% NaCl, to a final volume of 200 mL. The pharmacy added a blinded label to the infusion bag before distribution to the site. Participants and all study personnel were blinded. Monitoring of medication and randomization list handling was performed by an independent Good Clinical Practice (GCP) monitor.

Ghrelin was administered continuously as an IV infusion of 5 pmol/kg/min (16.9 ng/kg/min) for up to 4 hours. The dose was based on body weight and multiple published studies, including a landmark study demonstrating that 5 pmol/kg/min (compared with placebo, or 1 pmol/kg/min) reproduced the physiological effects of ghrelin, including a robust induction of appetite (21). Because i.v. ghrelin takes about 60 minutes to reach steady state and approximately 120 minutes to reach its full effect, fMRI scanning was carried out between 120–180 minutes after infusion start. No sex adjustment was used, since previous studies had indicated that endocrine effects of i.v. ghrelin are independent of sex (41, 42). Infusions were administered with an MR-compatible pump. Participants received up to 4.1 μg/kg during maximally 4 hour–long infusions.

### MID task

The MID task is widely used to assess reward processing in both healthy and clinical populations (15–17, 43). Participants are presented with geometrical shapes as cues representing different outcomes: circles (reward), squares (loss), and triangles (no loss/no reward). Rewards and losses vary in amount and can be low (± 10 Swedish krona [SEK] ≈ $1) or high (± 30 SEK ≈ $3), as represented by the number of lines on the circles and squares. To win as much money as possible, participants have to press a button when they are presented with a target (white square), either to gain rewards or avoid losses associated with the preceding cues. Participants familiarize themselves with the cues and the task structure beforehand, and the target interval is individually adapted to participants’ mean reaction times (RT) after a practice run outside the scanner, in order to achieve a 66% success rate [approximately 250 ms; (15)]. If the target is missed, actual money is either lost (punishment) or not gained (no reward), depending on the condition.

In our version (Supplemental Figure 2A), participants could monitor their own performance and a running total of their current gains on the screen during feedback on each trial. Participants were informed that they would start with 30 SEK and that the total amount (based on performance) at the end would be added to their actual compensation after each session (for a maximum SEK 430, approximately $43). Task duration was about 6 minutes, for a total of 50 trials. The task was written and presented with Presentation v17.2 (Neurobehavioral Systems Inc.). As stated, the target response interval was adapted to each participant’s baseline RT prior to scanning.

### Delay discounting task

Participants completed a computerized delay discounting task as described previously (20). In brief, they were repeatedly required to choose between amounts of money available that day (0–1000 SEK, in increments of 100) and 1000 SEK available after a delay of 0, 7, 30, 90, 180, 365, or 1,825 days (5 years), for a total of 76 trials. Participants were informed that 1 trial would be selected at random for compensation, and that they would receive the sum of money on that trial either now (after the session) or later, with the actual delay depending on what they chose. All combinations of immediate reward and delay interval were presented in a pseudorandomized order, and included the 1,000 SEK–now versus 1,000 SEK–later trials as an attention check.

For each delay interval, a “switch point” was defined as the midpoint between the lowest immediate reward selected by the subject and the next lowest immediate reward in the sequence (i.e., the value of immediate reward at which the subject began consistently to select the standard 1,000 SEK delayed reward), and was determined by fitting choice data to a square-wave function. We chose this fitting procedure because it was relatively robust and stood up well to occasional inconsistencies in participants’ choice behavior. The 7 indifference points were then fitted to a hyperbolic discounting function *V* = A*[1/(1 + k*D)], where *V* is the indifference value, *A* is the fixed 1000 SEK delayed reward, *D* is the delay in days, and *k* is the discounting coefficient. Higher values of *k* indicate a steeper discounting of reward value as function of time, i.e. higher preference for immediate reward; lower *k* values reflect less steep discounting, and a higher relative preference for delayed reward. We used MATLAB (The MathWorks Inc.) for nonlinear curve fitting. We calculated an *R*^2^ value for each subject to indicate goodness-of-fit; the median *R*^2^ was 0.86, similar to what has been observed previously using this task (20). Analyses were repeated measures ANCOVAs with intervention as a within subject factor, subject as a random factor, and sex, age, BMI, and session order as covariates. *P* ≤ 0.05 was considered significant. For Posthoc analysis, Newman-Keuls tests were used.

### MRI

#### Image acquisition.

Image acquisition was performed with a Siemens Prisma 3T scanner (Siemens Healthcare Gmbh), using a 64-channel head coil. Functional, blood-oxygen level-dependent (BOLD) T2*-weighted data were acquired with an echo-planar imaging (EPI) sequence (repetition time = 878 ms; echo time = 24 ms; flip angle = Ernst angle (56°); field of view = 476 × 476 mm; in plane resolution = 3 × 3 mm, slice thickness = 3 mm, parallel imaging factor = 1, simultaneous multi-slice factor = 3). For precise anatomical localization of functional effects, a high-resolution anatomical 3D T1-weighted MPRAGE scan was acquired before EPI data acquisitions (repetition time = 2,300 ms; echo time = 2.36 ms; flip angle = 8°; field of view = 288 × 288 mm; voxel resolution = 0.87 × 0.87 × 0.90 mm).

Participants were equipped with MR-compatible goggles for stimulus presentation in the scanner. After the initial T1 sequence, a 12-minute resting state BOLD scan was obtained, during which participants were asked to keep their eyes open and focus on a fixation cross. They then performed tasks in a counterbalanced order. The order was the same on both sessions. The total scan time was no more than 90 minutes.

#### Preprocessing.

Preprocessing and formal analysis of functional task data was performed in Analysis of Functional Neuroimages (AFNI) software version 21.2.08 (44), with preprocessing steps based on current AFNI recommendations for task-based fMRI (https://afni.nimh.nih.gov/pub/dist/doc/program_help/afni_proc.py.html). A whole-brain, voxel-wise GLM analysis was used to model BOLD time-series task data, using the AFNI 3dDeconvolve function. BOLD images were despiked and slice time corrected. EPI volume was registered to the volume with minimum outlier fraction and then warped to MNI template space, using affine and nonlinear transformations. Images were blurred with a 4 mm full width at half-maximum (FWHM) filter. Motion parameters were included as a regressor of no interest to factor out noise. A threshold of 0.2 mm between repetition times (TRs) was set as a motion censoring criterion, and an outlier fraction threshold of 0.05 was also used.

#### MID task.

First-level analyses included regressors modelling the conditions of anticipation and feedback, resulting in a total number of 11 regressors. Three anticipation regressors were defined as the onset of the cues associated with either reward (high/low), loss (high/low), or neutral (no loss/no reward) until the onset of the target. Feedback was modelled using onset-to-offset of the outcome presented on each trial. Loss and reward feedback regressors were collapsed over high and low amounts to increase the number of stimuli per regressor. Neutral feedback was represented by the outcome of the neutral no loss/no reward cue (± 0 SEK). Regressors representing losses as punishments and missed rewards as nonrewards were used to model unsuccessful trials with respect to behavioral performance. A motor regressor was also included to model button presses related to the task. Correction for multiple comparisons of group-level data was performed with 3dClustSim (45). Spatial group smoothness parameters were estimated based on first-level residuals, with censored TRs removed, and entered into a simulated gray matter group mask of the assembled EPI masks of participants who were not censored. Two subjects were excluded from statistical analysis due to medium degree censoring (> 25% of TRs censored), leaving *n* = 28 for second-level analysis. The 3dFWHMx function was used to compute the average spatial smoothness estimates from the residual maps, using a nongaussian autocorrelation function (ACF). The group mask was then multiplied with an MNI gray matter template mask to specifically target gray matter voxels for statistical analysis. As per current best practice (45, 46), alphas were set to *P* = 0.002 per voxel, and *P* = 0.05 at cluster level, family wise error (FWE) corrected. A minimum cluster size of 11 voxels was determined accordingly (bi-sided, nearest neighbor=1; voxel faces must touch).

Group-level analyses were run using AFNI 3dMVM (47). All MVM analyses were run as factorial ANOVAs. To identify the striatal cluster responsive to value anticipation, we included a single anticipation factor. This was based on a recent meta-analysis that established a generalized neural system, within which overlapping activations are associated with value-based motivational processes, irrespective of valence (17). We confirmed that this was replicated in our data, where activations associated with reward and loss anticipation overlapped, and showed the same response pattern to variation in reward and loss levels (Supplemental Figures 4–8). For our main MVM analysis, we therefore used a single anticipation regressor that spanned all value levels used in the task (high loss – low loss – neutral – low reward – high reward).

The objective of the whole-brain MVM analysis was to empirically delineate the anticipation-responsive striatal cluster in our data, rather than relying on a literature-based region-of-interest (ROI). To properly account for the within-subject design of our study, we also included intervention (ghrelin versus placebo) as a factor in the whole-brain analysis. The main effect of this factor was not of interest for identifying the anticipation-responsive cluster, while the power required to detect an anticipation × intervention interaction in the whole brain analysis vastly exceeds that for detecting main effects. To test for effects of ghrelin on striatal activations during anticipation, the whole brain analysis was therefore followed up by extracting β coefficients from the striatal cluster identified by the anticipation factor, and analyzing these β coefficients using factorial ANOVAs. These analyses were performed in SPSS version 28 (IBM). Several sensitivity analyses supporting the robustness of our approach are presented in Supplemental Figures 4–8.

Biological sex (male/female) was included as a covariate in all analyses. Sensitivity analyses were also carried out to examine potential contributions from age and BMI as covariates, but their inclusion did not change the results, and they were therefore dropped in the final analysis. The order of active ghrelin challenge and placebo was balanced across subjects in the randomization and was therefore not included as a factor.

#### Delay discounting.

For first-level, subject-wise analysis of the fMRI data we constructed 2 regressors of interest. The less-now regressor reflected γ-function-convolved δ functions during decision epochs when subjects decided to take less money made available that day. The more-later regressor reflected times at which subjects decided to take more money made available at the delay interval for that trial. The decision epoch for each trial was defined as the time from when choice stimuli were presented to when a response was made. The 2 regressors of interest spanned just this period of time. Response and feedback epochs for each trial were also modeled as regressors of no interest as were trials for decisions directly proximate to the indifference point. Pilot testing showed that clearer neural-functional effects were obtained by exclusion of these borderline decision trials.

Group-level analyses were run using a similar 3dMVM approach as the MID task, using a factorial ANOVA with intervention (placebo/ghrelin) and choice (less-now/more-later) as 2 within subject factors. Furthermore, we examined relations between *k* values obtained during a given session (ghrelin or placebo) both as main effects and interacting with choice. Based on results from analysis of the behavioral data for the delay discounting task, we analyzed the imaging data both including sex as a covariate of interest and in females and males independently. Similar to the MID, age, and BMI were evaluated as potential covariates, but did not affect the results, and were dropped from the final analysis. To correct for family wise type–1 error we used a per-voxel statistical threshold of *P* = 0.05 coupled with a minimum cluster size of 166 voxels (bisided, nearest neighbor=1).

### Plasma analyses

At each time point (Figure 1A), peripheral venous blood was collected into 6 mL EDTA tubes. Plasma concentrations of the following hormones were measured: ghrelin (referring to the active form, i.e., acyl-ghrelin), total ghrelin, growth hormone (GH), leptin, glucagon-like peptide-1 (GLP-1), pancreatic polypeptide (PP), gastric inhibitory peptide (GIP), peptide tyrosine tyrosine (PYY), and cortisol. Cortisol and growth hormone (GH) was analyzed at the Linköping University Hospital Clinical Chemistry laboratory (Laboratoriemedicin, Universitetssjukhuset i Linköping). Blood samples for analysis of the remaining hormones were centrifuged as soon as possible and within 30 minutes after collection (1,700*g*, 4°C, 15 minutes). The plasma supernatant was aliquoted in portions of 500 μL and stored at −80°C until analysis. A protease inhibitor Pefabloc SC (AEBSF; Roche) was added to inhibit degradation of ghrelin before blood collection and samples were acidified with HCl to a final concentration of 0.05 N after centrifuging, according to the manufacturer’s instructions.

Total ghrelin was measured using Human (Total) Ghrelin Kit (EMD Sigma-Aldrich). The kit has a sensitivity of 30 pg/mL in a 20 μL sample size, and samples were run in duplicates. During measurement of total ghrelin, samples outside the quantification range of the standard curve were further diluted with assay buffer in accordance with protocol and remeasured.

The Millipore Human Metabolic Hormone Magnetic Bead Panel 96-Well Plate MILLIPLEX_MAP_ kit (Sigma-Aldrich) was used to measure the following analytes within their respective reportable Minimum Detectable Concentration (MinDC): ghrelin (referred to as active ghrelin by the manufacturer), 13 pg/mL, leptin 41 pg/mL, total amylin 13 pg/mL, total GLP-1 2.5 pg/mL, PP 2 pg/mL, GIP 0.6 pg/mL, and PYY 28 pg/mL. The assay was performed on fluorescence-coded magnetic beads coated with capture antibodies specific for each marker. Introduction of biotinylated detection antibody and streptavidin-phycoerythrin permitted simultaneous detection of all analytes on a FLEXMAP 3D instrument (Luminex Corporation). These multiplex data were preprocessed and analyzed using MILLIPLEX Analyst software (Sigma-Aldrich) to calculate the concentration of each neuroendocrine marker. Intra- and interassay %CVs for all MILLIPLEX analytes included in the analysis were < 10% and < 15%, respectively, as determined by the manufacturer.

### Study approval

The study was carried out according to GCP, approved by the Swedish Ethics Review Authority (Dnr. 2019-01510) and the Swedish Medicinal Products Agency, and preregistered as EudraCT 2018-004829-82. Data for secondary analyses will be made available with a transfer agreement.

### Statistics

Sample size was chosen based on a priori power analysis, to achieve adequate power for detecting a moderate or greater effect size (Cohen’s d ≥ 0.6) on the predefined primary outcomes at α = 0.05, based on prior studies (reviewed in ref. 12). Dropouts were replaced. Thirty participants completed the study. For whole brain analyses of fMRI data, General Linear Models were used as implemented in the AFNI software package and are described in detail in Methods. For other statistical analysis, ANOVA followed by Newman-Keul’s post-hoc tests was used, with design factors, their within- or between subjects nature, and covariates provided in Results and figure legends. All reported *P* values are 2 tailed. *P* values of less than 0.05 were considered significant.

## Author contributions

MH, GT, LL, and MP conceptualized the project. MH, LL, JPH, IP, RK, RB, and EP chose and developed the methodology. MP, SG, AY, AA, EG, and AL conducted the investigation. MH and GT acquired funding for the project. MH and AJC supervised the project. MP, AY, MH, and GT wrote the manuscript. MH and GT jointly and in equal part conceptualized, planned for, and funded the study, under a mutual a priori agreement to share senior authorship. MP led the work on implementation and execution of the live phase of the study, while AY led the work on assembling the manuscript, contributions that the entire team agreed were equal.

## Supplementary Material

Supplemental data

ICMJE disclosure forms

## Version 1 04/11/2023

In-Press Preview

## Version 2 06/15/2023

Electronic publication

## References

[1] Muller TD (2015). Ghrelin. Mol Metab.

[2] Kenny PJ (2011). Common cellular and molecular mechanisms in obesity and drug addiction. Nat Rev Neurosci.

[3] Farokhnia M (2019). Ghrelin: From a gut hormone to a potential therapeutic target for alcohol use disorder. Physiol Behav.

[4] Zallar LJ (2017). The role of the ghrelin system in drug addiction. Int Rev Neurobiol.

[5] Jerlhag E (2009). Requirement of central ghrelin signaling for alcohol reward. Proc Natl Acad Sci U S A.

[6] Rangel A (2008). A framework for studying the neurobiology of value-based decision making. Nat Rev Neurosci.

[7] Danziger S (2011). Extraneous factors in judicial decisions. Proc Natl Acad Sci U S A.

[8] Glöckner A (2016). The irrational hungry judge effect revisited: Simulations reveal that the magnitude of the effect is overestimated. Judgm Decis Mak.

[9] Persson E (2019). The effect of decision fatigue on surgeons’ clinical decision making. Health Econ.

[10] Anderberg RH (2016). The stomach-derived hormone ghrelin increases impulsive behavior. Neuropsychopharmacology.

[11] Ralevski E (2018). Ghrelin is related to personality differences in reward sensitivity and impulsivity. Alcohol Alcohol.

[12] Garin MC (2013). Clinical review: The human experience with ghrelin administration. J Clin Endocrinol Metab.

[13] Leggio L (2014). Intravenous ghrelin administration increases alcohol craving in alcohol-dependent heavy drinkers: a preliminary investigation. Biol Psychiatry.

[14] Farokhnia M (2018). Exogenous ghrelin administration increases alcohol self-administration and modulates brain functional activity in heavy-drinking alcohol-dependent individuals. Mol Psychiatry.

[15] Knutson B (2001). Anticipation of increasing monetary reward selectively recruits nucleus accumbens. J Neurosci.

[16] Jauhar S (2021). Brain activations associated with anticipation and delivery of monetary reward: A systematic review and meta-analysis of fMRI studies. PLoS 1.

[17] Oldham S (2018). The anticipation and outcome phases of reward and loss processing: A neuroimaging meta-analysis of the monetary incentive delay task. Hum Brain Mapp.

[18] Schott BH (2008). Mesolimbic functional magnetic resonance imaging activations during reward anticipation correlate with reward-related ventral striatal dopamine release. J Neurosci.

[19] Knutson B, Gibbs SE (2007). Linking nucleus accumbens dopamine and blood oxygenation. Psychopharmacology (Berl).

[20] Hariri AR (2006). Preference for immediate over delayed rewards is associated with magnitude of ventral striatal activity. J Neurosci.

[21] Wren AM (2001). Ghrelin enhances appetite and increases food intake in humans. J Clin Endocrinol Metab.

[22] Seghier ML (2013). The angular gyrus: multiple functions and multiple subdivisions. Neuroscientist.

[23] Humphreys GF (2021). A unifying account of angular gyrus contributions to episodic and semantic cognition. Trends Neurosci.

[24] Ramanan S (2018). Rethinking the role of the angular gyrus in remembering the past and imagining the future: the contextual integration model. Neuroscientist.

[25] Han JE (2018). Ghrelin enhances food odor conditioning in healthy humans: an fMRI study. Cell Rep.

[26] Dickson SL (2011). The role of the central ghrelin system in reward from food and chemical drugs. Mol Cell Endocrinol.

[27] Mason BL (2014). The central nervous system sites mediating the orexigenic actions of ghrelin. Annu Rev Physiol.

[28] Abizaid A (2006). Ghrelin modulates the activity and synaptic input organization of midbrain dopamine neurons while promoting appetite. J Clin Invest.

[29] Volkow ND (2019). The neuroscience of drug reward and addiction. Physiol Rev.

[30] Weinberg ZY (2011). 6-Hydroxydopamine lesions of the ventral tegmental area suppress ghrelin’s ability to elicit food-reinforced behavior. Neurosci Lett.

[31] Sztainert T (2018). Hungry to gamble? Ghrelin as a predictor of persistent gambling in the face of loss. Biol Psychol.

[32] Poulton A, Hester R (2020). Transition to substance use disorders: impulsivity for reward and learning from reward. Soc Cogn Affect Neurosci.

[33] Bernardoni F (2020). Metabolic state and value-based decision-making in acute and recovered female patients with anorexia nervosa. J Psychiatry Neurosci.

[34] McClure SM (2004). Separate neural systems value immediate and delayed monetary rewards. Science.

[35] Ballard K, Knutson B (2009). Dissociable neural representations of future reward magnitude and delay during temporal discounting. Neuroimage.

[36] Nee DE (2007). Interference resolution: insights from a meta-analysis of neuroimaging tasks. Cogn Affect Behav Neurosci.

[37] Rusconi E (2018). Gerstmann syndrome: historic and current perspectives. Handb Clin Neurol.

[38] Sozou PD (1998). On hyperbolic discounting and uncertain hazard rates. Proc R Soc Lond B Biol Sci.

[39] Weafer J, de Wit H (2014). Sex differences in impulsive action and impulsive choice. Addict Behav.

[40] Smith A (2022). Ghrelin and the control of energy balance in females. Front Endocrinol (lausanne).

[41] Broglio F (2003). The endocrine response to ghrelin as a function of gender in humans in young and elderly subjects. J Clin Endocrinol Metab.

[42] Levin F (2006). Ghrelin stimulates gastric emptying and hunger in normal-weight humans. J Clin Endocrinol Metab.

[43] Knutson B, Heinz A (2015). Probing psychiatric symptoms with the monetary incentive delay task. Biol Psychiatry.

[44] Cox RW (1996). AFNI: software for analysis and visualization of functional magnetic resonance neuroimages. Comput Biomed Res.

[45] Cox RW (2017). FMRI clustering in AFNI: false-positive rates redux. Brain Connect.

[46] Cox RW (2017). fMRI clustering and false-positive rates. Proc Natl Acad Sci U S A.

[47] Chen G (2014). Applications of multivariate modeling to neuroimaging group analysis: a comprehensive alternative to univariate general linear model. Neuroimage.

