# Supplementary material for: A randomized controlled experimental medicine study of ghrelin in value-based decision making
Source: J Clin Invest. 2023 Jun 15;133(12):e168260. doi: 10.1172/JCI168260 (PMC10266781; doi:10.1172/JCI168260)
Supplement: Supplemental data [file jci-133-168260-s243.pdf]

SUPPLEMENTAL MATERIALS

Supplemental methods

Participant eligibility and disposition

Participants were included between 2020-03-13 and 2021-05-07 at the Center for Social and Affective Neuroscience, Linköping University Hospital, Linköping, Sweden. A CONSORT diagram showing disposition of participants is in **Fig. S1,** and an overview of study visits in **Table S1.**

Inclusion criteria:

1. Age 18-65 years.
2. Good health as determined by medical history, physical exam, ECG (electrocardiogram), and clinical assessment of lab tests.
3. Females provided a negative urine pregnancy (hCG; human chorionic gonadotropin) test at the start of each study session.

Exclusion criteria:

1. Any clinically significant medical condition, including CNS (central nervous system), cardiovascular, respiratory, gastrointestinal, hepatic, renal, endocrine, or reproductive disorders.
2. Specific exclusion criteria related to the administration of ghrelin were chronic inflammatory diseases (e.g., Crohn's disease, ulcerative colitis, celiac disease), diabetes, obesity (Body Mass Index ≥ 30 kg/m^2), weight ≥ 120 Kg, high triglycerides level (> 350 mg/dL), history of clinically significant hypotension (e.g. history of fainting and/or syncopal attacks) and/or resting systolic blood pressure < 100 mmHg.
3. Any current clinically significant psychiatric problems; any history of a past eating disorder, psychotic illness, or bipolar disorder; as determined by medical history, clinical examination and MINI interview carried out by appropriately trained staff.
4. Current diagnosis of substance dependence other than nicotine.
5. Inability to provide a negative urine drug screen.
6. Pregnancy, intention to become pregnant, or breastfeeding a child.
7. Contraindications for MRI scanning.

Randomization

Randomization and blinding were independent of study personnel, by Forum Östergötland, the clinical trials service of Linköping University Hospital, and the hospital pharmacy. Because the study was a within-subject design, only the sequence of sessions (ghrelin followed by placebo, vs. placebo followed by ghrelin) was randomized, stratified for sex, using pre-generated random number lists.

Supplemental results

Monetary Incentive Delay (MID) task

Reaction Time and Number of hits

Reaction times did not differ between ghrelin and placebo sessions, neither overall (grand mean: 223.7±2.2 vs. 226.1±2.3, mean±SEM; paired t-test: p=0.26), nor within any individual trial category [RM-ANOVA:s with Intervention (ghrelin vs. placebo) as within subject factor, and session order and sex as covariates: reward trials: p=0.12; loss trials: p=0.26; neutral trials: p=0.19].

Similarly, the total number of hits did not differ between ghrelin and placebo sessions, neither overall (grand mean: 43.6±0.64 vs. 43.0±0.67; p=0.47), nor within any individual trial category [RM-ANOVA:s with Intervention (ghrelin vs. placebo) as within subject factor, and session order and sex as covariates: reward trials: p=0.48; loss trials: p=0.12; neutral trials: p=0.57].

Sensitivity analyses – Intervention x Outcome anticipation whole-brain analysis

Compared to the MVM model controlling for sex reported in the main text (Figure 2), additional controls for BMI and age (also controlling for sex) only marginally reduced the main effect of Anticipation on striatal activity from 1734 voxels to 1689 voxels, using the same Intervention (placebo/ghrelin) x Anticipation (high loss/low loss/neutral/low reward/high reward) approach, and no main effects of either age nor BMI (or sex) were identified.

Reward anticipation

Whole-brain MVM analysis of the anticipation phase showed the expected main effect of reward anticipation [F(1,40)=45.91, p<0.001, ηp^2=0.63], with a large cluster in the ventral striatum (1188 voxels). Ventral striatal activation was proportional to reward magnitude, with significantly higher activation for maximum reward anticipation vs lower reward and neutral anticipation (all ps<0.001; Bonferroni corrected). Low reward also resulted in increased activity vs neutral anticipation (mean difference ± SEM: 0.039±0.007, p<0.001), illustrating that striatal activity scaled with reward magnitude (Figure S4). Activity in this cluster was unaffected by ghrelin (main effect: p=0.49; interaction: p=0.77). Sex did not have any significant effects on the results.

The specific analysis of activations associated with losses rather than rewards is provided in the next section. However, we noted that, when analyzing extracted betas within the cluster identified by the analysis of reward anticipation, there was also a robust main effect of loss anticipation [F(2,54)=19.10, p<0.001, ηp^2=0.41]. This effect showed a similar parametric increase for each amount, with the highest activation for high loss compared to lower loss (mean difference ± SEM:

0.019±0.007, $p=0.026$) and neutral (mean difference ± SEM: 0.048±0.009, $p<0.001$). Lower loss anticipation was also significantly different from neutral (mean difference ± SEM: 0.029±0.008, $p=0.003$). We also found a main effect of Intervention on loss anticipation in this cluster [$F(1,27)=5.56$, $p=0.026$, $\eta p^2=0.17$], with stronger deactivation during ghrelin compared to placebo (mean difference ± SEM: 0.019±0.008, $p=0.026$). As shown in Figure S4, there was a trend towards an Intervention x Anticipation interaction ($p=0.091$), illustrating the parametric nature of the relationship. Sex did not have a significant effect.

Further inspection of the whole-brain analysis also revealed a small main effect of Intervention, with higher activation in the right calcarine gyrus during ghrelin compared to placebo (see Figure S10).

Loss Anticipation

When activations associated with loss anticipation were specifically analyzed, we found striatal activations similar to those observed during reward anticipation (see Figure S5), with the largest striatal cluster in the right putamen (262 voxels). A main effect of Anticipation [$F(2,54)=32.84$, $p<0.001$, $\eta p^2=0.55$] revealed a similar parametric increase in activation for losses, with significantly higher activation for maximum loss compared to low loss (mean difference ± SEM: 0.030±0.008, $p<0.001$) and neutral anticipation (mean difference ± SEM: 0.057±0.008, $p<0.001$). Low loss activation was significantly higher compared to neutral (mean difference ± SEM: 0.028±0.007, $p=0.001$). There was no main nor interaction effect of Intervention ($p=0.147$ and $p=0.458$, respectively). Sex did not have any significant effects.

Similar to the ventral overlap between loss and reward, there was an increase in reward activity in this dorsal cluster, with a strong main effect of Anticipation [$F(1,38)=30.74$, $p<0.001$, $\eta p^2=0.53$], showing a significantly higher activation for high reward compared to low reward (mean difference ± SEM: 0.016±0.005, $p=0.02$) and neutral (mean difference ± SEM: 0.056±0.009, $p<0.001$), while low reward also was significantly different compared to neutral (mean difference ± SEM: 0.040±0.007, $p<0.001$). There was no main nor interaction effect of Intervention ($p=0.835$ and $p=0.268$, respectively). There was no effect of Intervention. Sex did not influence the results.

Only two small additional clusters influenced by Intervention were identified across the whole brain, with stronger deactivation of the left postcentral gyrus (13 voxels) and increased activation in the right calcarine gyrus (12 voxels) during ghrelin compared to placebo (Figure S11).

Feedback phase

During feedback, the MVM results showed a significant Intervention x Feedback interaction, F(1, 26)=9.63, p=0.005, ηp^2=0.27, revealing a (whole-brain corrected) cluster of bilateral activation in the right posterior MCC [MNI:2, 14, 41]. Compared to placebo, ghrelin elicited increased posterior MCC activation in response to successfully obtained rewards relative to neutral feedback (mean difference ± SEM: 0.123±0.048, p=0.016; Bonferroni corrected). MCC activation in response to reward was also coupled to a stronger deactivation in response to neutral feedback during the ghrelin infusion.

Motion Analyses

A paired t-test showed a numerically small but statistically significant difference in average motion per TR between the placebo and ghrelin session (mean difference: 0.007 mm, p=0.034; placebo > ghrelin). Closer inspection showed that this was mainly driven by females (p=0.044; placebo > ghrelin), while average motion per TR was not significantly different between sessions in males (p=0.15). Max motion displacement and censor fraction per stimulus did not differ between the two sessions overall (p=0.22 and p=0.077, respectively). At trend-level, censor fraction per stimulus was slightly higher during placebo, mainly driven by two subjects with 27% and 35% of stimuli censored. These subjects were removed from all statistical analyses.

Delay Discounting (DD) Task

Average response time did not differ between ghrelin and placebo sessions [1.87s±0.06 vs. 1.84±0.05, mean±SEM; RM-ANOVA with Intervention as within subject factor and session order and sex as covariates, main effect of Intervention: F(22,1,1)=0.56; p=0.56]. Similarly, there was no Intervention effect on reaction time during less-now easy choices (p=0.98), less-now hard choices (p=0.87), more-later easy choices (p=0.77) or more-later hard choices (p=0.74).

Adverse events

No treatment-emergent adverse events were recorded. The adverse events recorded were all mild, transient, related to the inconvenience of MR scanning, and evenly distributed between ghrelin and placebo sessions. Please see **Table S7** for all reported side effects.

Participant characteristics

Neither age nor BMI differed between men and women, as confirmed by independent samples t-test (p=0.64 and p=0.66, respectively; see **Table S8** for mean and SEM values).

CONSORT 2010 Flow Diagram

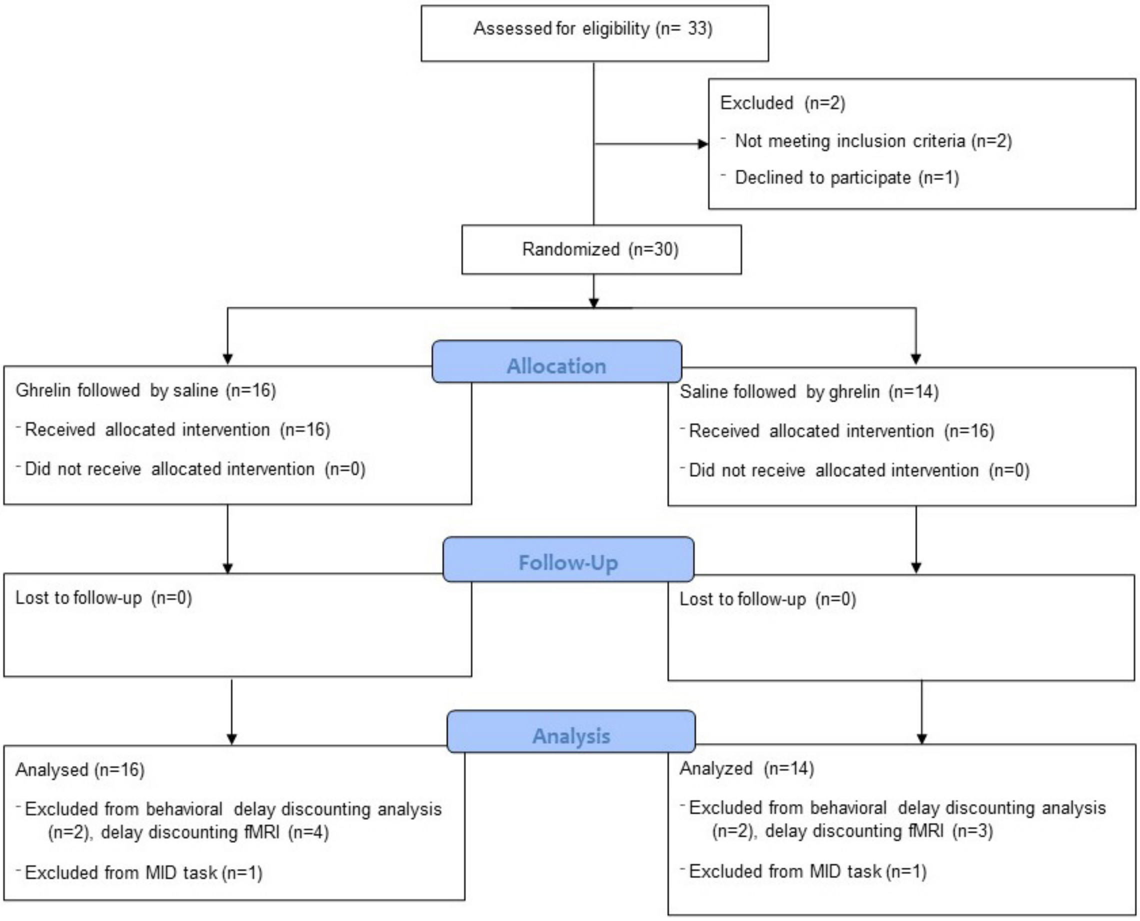

Fig. S1. CONSORT diagram

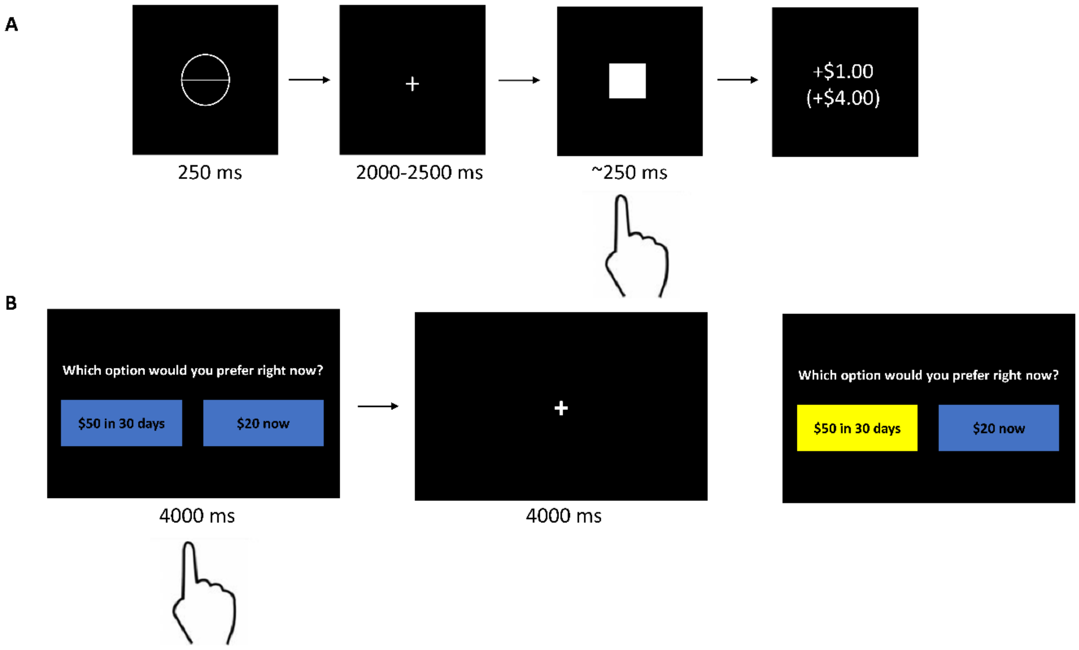

Fig. S2. Images of fMRI task screens. Actual images were in Swedish, and provided the corresponding amounts in Swedish Krona (SEK). **A)** MID task. From left to right: Stimulus presentation indicating the valence and magnitude of the prospective outcome, as practiced prior to the scan; anticipation phase; response; and feedback. **B)** Delay Discounting task. From left to right: Stimulus presentation indicating the value and temporal distance of the available options; anticipation phase; feedback phase.

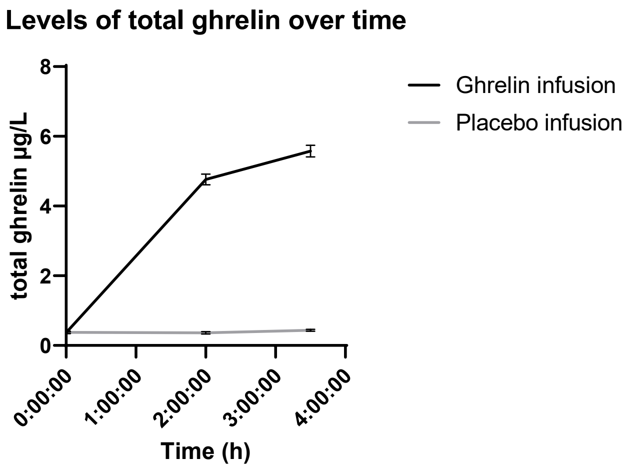

Fig. S3. Plasma ‘total ghrelin’ levels resulting from the infusion. Data points are mean+SEM; n=30

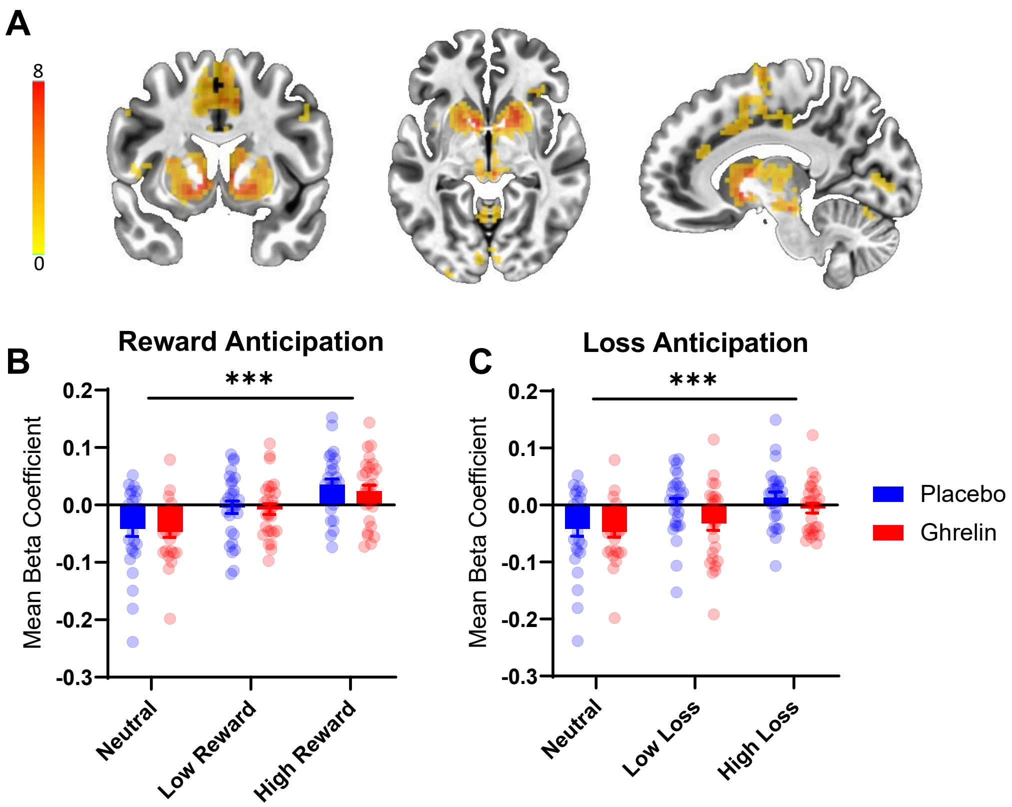

Fig. S4: **A)** Activation map showing the peak of the cluster associated with reward anticipation in the MID task [MNI: -2, 1, 1] and **B)** bar graph showing main effect of Reward Anticipation [F(1,40)= 45.91, p<0.001, ηp^2=0.63], but no significant main effect of Intervention (p=0.49) nor interaction (p=0.68. **C)** Main effect of Loss Anticipation in the same cluster [F(2,54)=19.10, p<0.001, ηp^2=0.41], and a main effect of Intervention [F(1,27)=5.56, p=0.026, ηp^2=0.17), including a trend for an Intervention x Anticipation interaction (p=0.091). Error bars denote SEM. Colored panel represents T statistic.

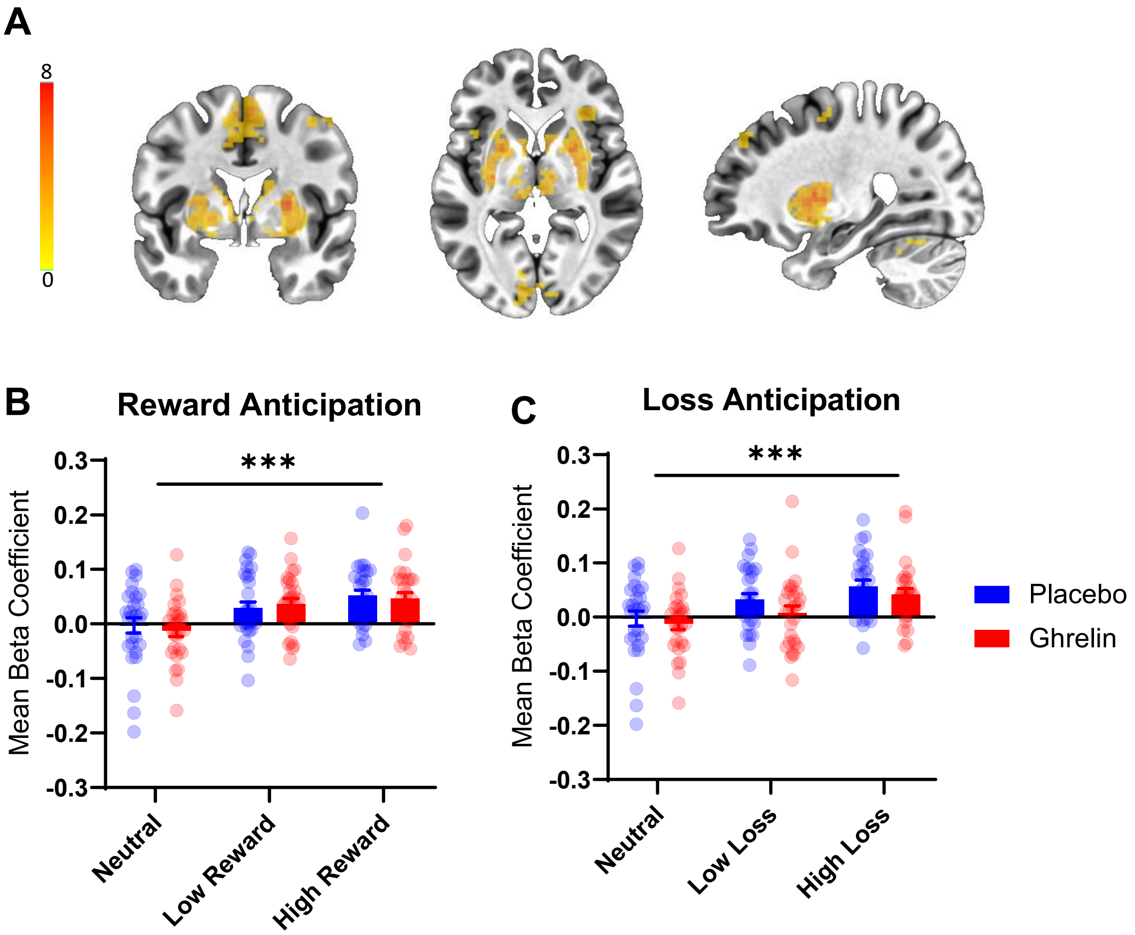

Fig. S5: A) Activation map of the cluster associated with loss anticipation in the MID task [MNI: 26, 1, 1]. B) *** denotes main effect of Reward Anticipation [$F(1,38)=30.74$, $p<0.001$, $\eta p^2=0.53$]. There were no effects of Intervention. **C)** *** denotes main effect of Loss Anticipation [$F(2,54)= 32.84$, $p<0.001$, $\eta p^2=0.55$). Colored panel represents T statistic.

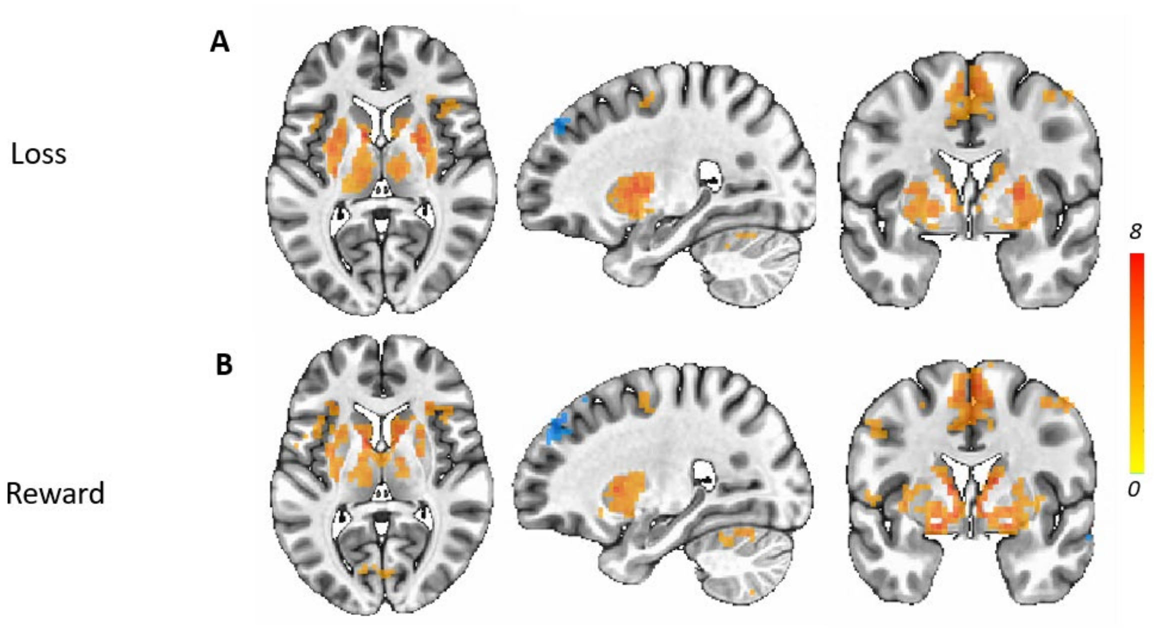

Fig. S6. Activation map with loss anticipation cluster [MNI: 26, 1, 1], demonstrating the overlap between **A)** loss anticipation, and **B)** reward anticipation in the dorsal striatum. Colored panel represents T statistic.

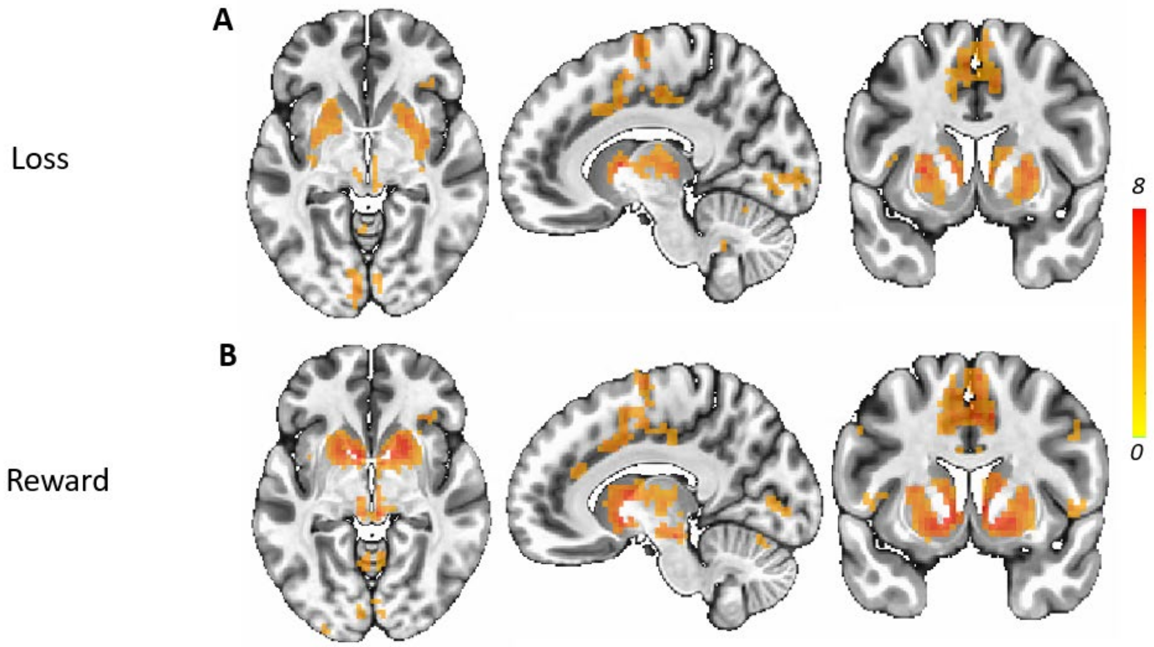

Fig. S7. Activation map with the reward anticipation cluster [MNI:-2, 1, 1], showing the overlap between **A)** loss anticipation, and **B)** reward anticipation in the ventral striatum. Colored panel represents T statistic.

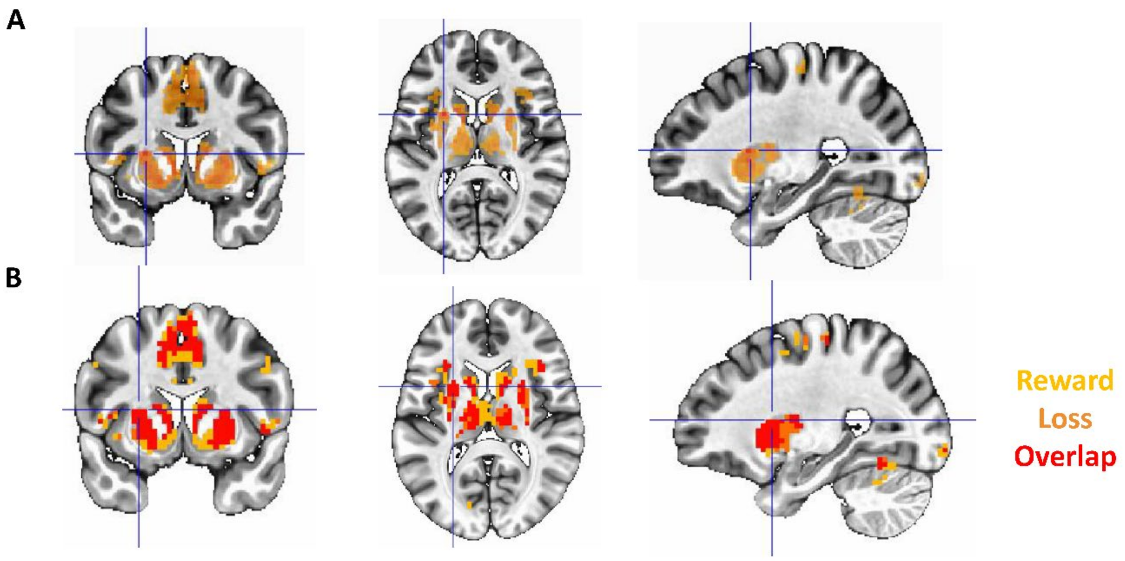

Fig. S8. Conjunction analysis confirmed the overlap of the reward- and the loss-sensitive clusters that provided the rationale for final analysis of ghrelin effect to be carried out on the concatenated cluster **A)** The concatenated cluster used for the final analysis, with activation peak indicated by the crosshair. **B)** Conjunction analysis indicating the overlap.

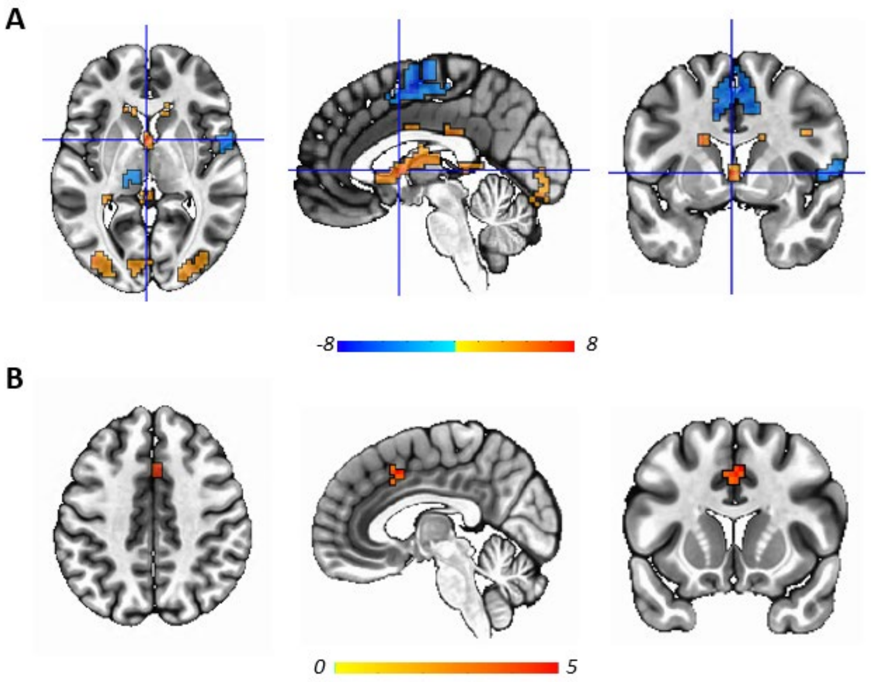

Fig. S9. A) Main effect of feedback (reward > neutral) in the MID task; **B**) Interaction Feedback x Intervention (ghrelin > placebo; reward > neutral). Colored panel represents T statistic.

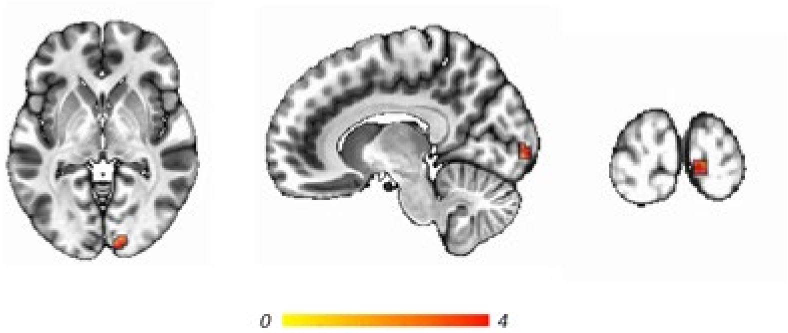

Fig. S10. Main effect of Intervention (ghrelin > placebo) on right calcarine gyrus activity [MNI: 13, -95, 0] during reward anticipation. Colored panel shows T statistic.

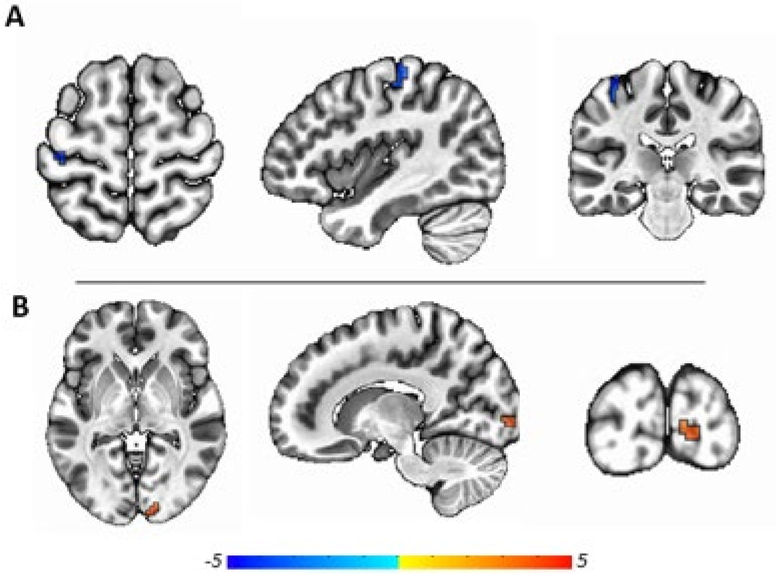

Fig. S11. Main effect of Intervention (ghrelin > placebo) during loss anticipation in A) left postcentral gyrus [MNI:-39, -26, 61], and B) right calcarine gyrus [MNI: 13, -95, 1]. Panel shows T statistic.

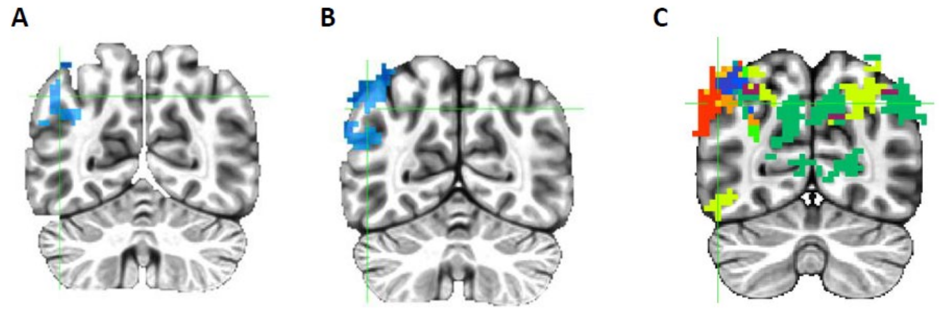

Fig. S12. Conjunction analysis confirmed the overlap of the clusters identified by the whole-brain analysis for activity associated with the hyperbolic discounting constant *k* under both placebo and ghrelin conditions. **A)** The cluster identified by the analysis for *k* under placebo conditions; **B)** Cluster identified by the analysis for *k* under ghrelin conditions **C)** Conjunction indicating the overlap between main effects of *k* during ghrelin and placebo in bright orange, with crosshairs in [-49, -69, 41].

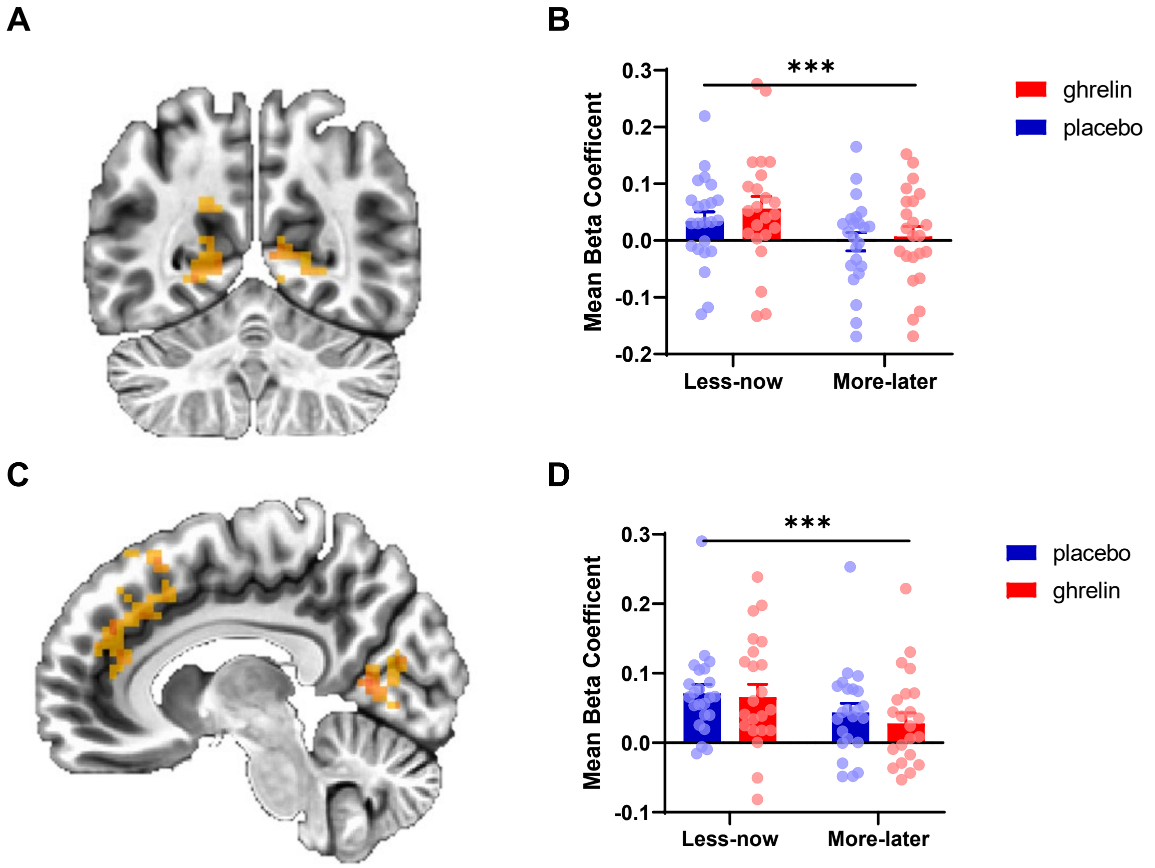

Fig. S13. Whole brain analysis for the effect of Choice identified areas with increased activity during less-now vs. more-later decisions in the left precuneus and bilateral calcarine gyri within primary visual cortex (**A, B**) **(B)** *** denotes main effect of Choice [$F(1,45)=27.6$, $p<0.001$, $\eta p^2=0.38$), but no effects of Intervention. This effect was also seen in right dorsal anterior cingulate cortex (dACC) extending to medial prefrontal cortex (mPFC; **C,D**). **(D)** Main effect of Choice [$F(1,45)=29.0$, $p<0.001$, $\eta p^2=0.39$), but no effect of Intervention.

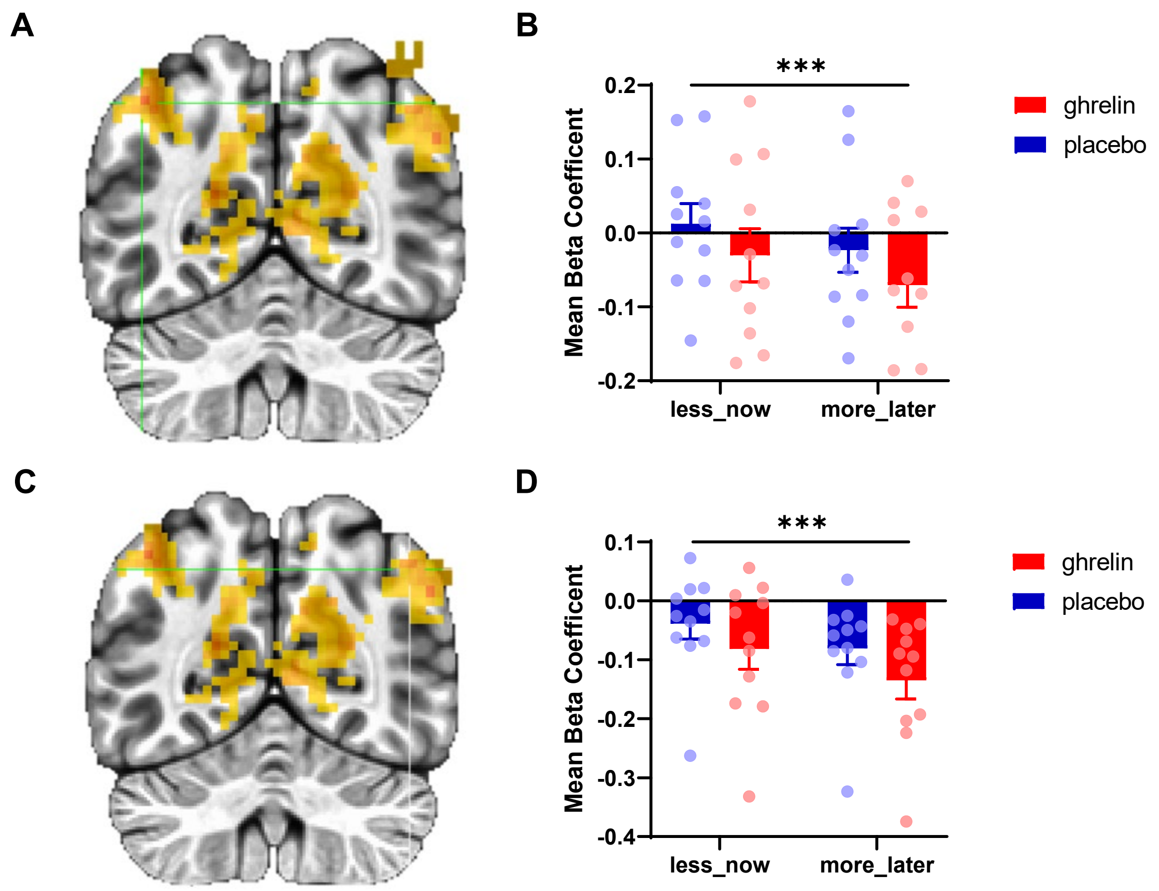

Fig. S14. When analysis for main effect of Choice was stratified by sex, additional areas were identified in females, including bilateral clusters in posterior parietal cortex that overlapped with those separately identified by the main effect of intervention. **(B)** *** denotes main effect of Choice [$F(1,21)=51$, $p<0.001$, $\eta p^2=0.71$] **(D)** *** denotes main effect of Choice [$F(1,21)=41.0$, $p<0.001$, $\eta p^2=0.66$].

Visit/Procedure	Visit 1 (screening)	Visit 2	Visit 3	Phone call 1-5 days after visit 3
Inclusion and exclusion	x	x		
Informed Consent Form	x			
hCG-test for women	x	x	x	
MINI, DUDIT,NEO-FF and AUDIT questionnaires	x			
Blood samples	x	x	x	
Randomization		x		
Weight of patient	x	x	x	
Length of patient	x			
Administration of Ghrelin/placebo		x	x	
fMRI incl. BOLD-data		x	x	
Adverse Events/Serious Adverse Events follow-up		x	x	x
End of the study				x

Table S1. Overview of study visits.

**Location**	**Cluster size**	**Center of mass MNI**
Cerebellum	2034	-2, -76, -10
Left striatum	1734	-2, 0, 1
Left SMA	836	-1, 0, 53
Left precentral gyrus	346	-37, -16, 58
Right angular gyrus	198	48, -62, 40
Right superior occipital gyrus	168	3, -70, 46
Right middle frontal gyrus	132	39, -4, 54
Right insula lobe	127	34, 24, 3
Left angular gyrus	89	-53, -61, 34
Right paracentral lobule	70	1, -35, 73
Left middle frontal gyrus	63	-39, 18, 55
Right cerebellum (VIII)	61	16, -64, -52
Right middle frontal gyrus	46	25, 44, 40
Right superior occipital gyrus	37	30, -75, 26
Right SMA	33	14, 18, 66
Left anterior cingulate cortex	27	-7, 30, 22
Right superior medial gyrus	26	16, 57, 32
Right superior medial gyrus	24	14, 34, 57
Left cerebellum (VII)	23	-32, -68, -55
Right middle frontal gyrus	23	45, 18, 49
Left postcentral gyrus	21	52, 0, 41
Right middle temporal gyrus	20	53, -69, -3
Right postcentral gyrus	20	20, -27, 66
Left angular gyrus	19	-40, -73, 46
Left superior frontal gyrus	19	-12, 32, 59
Right SMA	19	16, -14, 68
Left fusiform gyrus	18	-30, -38, -13
Right precentral gyrus	18	50, 5, 37
Right superior frontal gyrus	17	27, 27, 56
Left middle frontal gyrus	16	-28, 45, 40
Right cerebellum (X)	13	19, -39, -43
Left middle temporal gyrus	13	-56, 1, -32
Right anterior cingulate cortex	13	10, 35, 25
Right superior parietal lobule	13	28, -57, 52
Left cerebellum (X)	12	-20, -38, -43
Left inferior frontal gyrus	12	-50, 43, -15
Right superior occipital gyrus	12	15, -98, 20

Table S2. Activation locations for Anticipation (all reward and loss magnitudes and neutral) in the MID task. All regions surpassed the threshold for whole-brain significance, determined as described in Methods.

Location	Cluster size	Center of mass MNI
Left striatum	1188	-2, 1, 1
Left SMA	654	0, 2, 53
Cerebellum	445	9, 58, -23
Left precentral gyrus	191	-39, 13, 55
Left angular gyrus*	98	-49, -67, 36
Right middle frontal gyrus	91	39, -4, 54
Right insula	81	34, 26, 4
Left cerebellum (VI)	71	-32, -57, -24
Right inferior parietal lobule*	69	51, -62, 41
Right cerebellum (VIII)	68	17, -63, -51
Left middle frontal gyrus*	38	-38, 16, 55
Left calcarine gyrus	37	-8, -84, 2
Right superior frontal gyrus*	37	25, 44, 39
Right precuneus*	34	9, -56, 38
Right calcarine gyrus	28	5, -85, 1
Left precentral gyrus	22	-52, 1, 42
Brain stem	19	0, -31, -25
Right superior medial gyrus*	19	14, 34, 57
Brain stem	18	-1, -35, -38
Right superior medial gyrus*	18	15, 57, 31
Left inferior occipital gyrus	17	-25, -100, -8
Left middle occipital gyrus	16	32, -53, -51
Right cerebellum (VIII)	15	-30, -79, 24
Right precentral gyrus	15	50, 6, 38
Right middle frontal gyrus*	15	33, 25, 53
Left anterior cingulate cortex	14	-8, 32, 23
Left cerebellum (VI)	13	-10, -73, -17
Right middle temporal gyrus*	13	63, -4, -16
Right insula	12	30, 20, -10
Right middle frontal gyrus*	12	42, 15, 53
Right insula	11	50, 8, 2

Note. * = deactivation (high reward < neutral).

Table S3. Activation locations for Anticipation High reward > Neutral in the MID task. All regions surpassed the threshold for whole-brain significance, determined as described in Methods.

Location	Cluster size	Center of mass MNI
Left SMA	465	-1, -2, 55
Cerebellum	262	1, -59, -26
Right putamen	262	26, 1, 1
Left putamen	236	-26, 0, 2
Left caudate nucleus	168	-12, -10, 7
Left precentral gyrus	126	-41, -15, 54
Right thalamus	108	10, -17, 4
Left calcarine gyrus	65	-8, -89, -2
Right middle frontal gyrus	59	38, -3, 54
Right insula	53	35, 27, 3
Right caudate nucleus	52	11, 7, 8
Left calcarine gyrus	41	8, -83, -6
Brain stem	35	2, -32, -36
Right cerebellum (Crus 1)	32	34, -50, -30
Right paracentral lobule*	25	2, -36, 75
Right cerebellum (VIII)	21	14, -57, -35
Left insula	18	-41, 14, 6
Right superior frontal gyrus*	18	25, 44, 41
Left cerebellum (Crus 1)	13	-35, -53, -28
Left fusiform gyrus	13	-26, -60, -14
Left postcentral gyrus	13	-21, -28, 60

Note. * = deactivation (high loss < neutral).

Table S4. Activation locations for Anticipation High loss > Neutral in the MID task. All regions surpassed the threshold for whole-brain significance, determined as described in Methods.

Location	Cluster size	Center of mass MNI
Left SMA*	497	-1, -6, 59
Left lingual gyrus	461	-8, -83, -12
Right inferior occipital gyrus	340	40, -72, -5
Left precentral gyrus*	205	-30, -27, 62
Right caudate nucleus	199	1, 1, 7
Right superior occipital gyrus	105	30, -70, 38
Right inferior parietal lobule	88	45, -39, 45
Right postcentral gyrus*	84	23, -30, 66
Left middle occipital gyrus	74	-33, -88, 7
Right precentral gyrus*	57	43, -12, 53
Right rolandic operculum*	45	56, 3, 6
Left middle occipital gyrus	43	-28, -71, 32
Right middle frontal gyrus	40	43, 44, 16
Right precuneus*	38	9, -48, 60
Left caudate nucleus	30	-19, -6, 26
Right superior temporal gyrus*	28	50, -32, 23
Right postcentral gyrus*	26	30, -41, 62
Left middle frontal gyrus*	25	-37, 42, 33
Right caudate nucleus	24	-12, -24, 5
Left thalamus*	24	18, -13, 26
Left posterior cingulate cortex	23	0, -38, 6
Right anterior cingulate cortex	22	7, 36, 22
Left hippocampus	21	-33, -38, -1
Right inferior frontal gyrus	21	49, 10, 27
Right angular gyrus*	21	59, -55, 32
Left inferior parietal lobule*	19	-52, -58, 46
Left angular gyrus*	18	-56, -63, 37
Left inferior temporal gyrus	17	-46, -53, -13
Right hippocampus	15	36, -31, -8
Left insula	15	-19, -31, 17
Left cerebellum (VII)	14	-30, -75, -53
Right middle cingulate cortex	14	1, -29, 29
Left inferior parietal lobule	14	-47, -43, 45
Left postcentral gyrus*	13	-31, -42, 60
Left inferior parietal lobule*	12	-55, -23, 51
Right middle cingulate cortex	11	2, -5, 32

Note. * = deactivation (reward < neutral).

Table S5. Activation locations for Feedback Reward > Neutral in the MID task. All regions surpassed the threshold for whole-brain significance, determined as described in Methods.

		Region	Center of mass MNI coordinates	Cluster size in voxels	Description of effect
Full sample	Main effect of choice	Left precuneus and bilateral calcarine gyrus	-4, -70, 15	396	Increased activity for less-now versus more-later decisions
		Right dorsal anterior cingulate	8, 30, 36	173	Increased activity for less-now versus more-later decisions
	Main effect of intervention	Right superior and inferior parietal lobe / Right angular gyrus	35, -60, 48	248	Reduced activity for both less-now and more-later decisions in the ghrelin versus placebo sessions
		Left superior and inferior parietal lobe	-43, -48, 56	179	Reduced activity for both less-now and more-later decisions in the ghrelin versus placebo sessions
		Right middle frontal gyrus	39, 52, 5	177	Deactivation during ghrelin versus placebo sessions for both less-now and more-later decisions.
	Main effect of sex	Bilateral medial prefrontal cortex	-5, 47, 2	353	Deactivated selectively in women for both less-now and more-later decisions in both sessions.
		Left dorsomedial prefrontal cortex	-9, 45, 45	217	Deactivated selectively in women for both less-now and more-later decisions in both sessions.
		Left angular gyrus	-48, -62, 35	207	More deactivated in women for both less-now and more-later decisions in both sessions.
		Left middle temporal gyrus	-32, -80, 15	189	More activated in women for both less-now and more-later decisions in both sessions.
		Right declive of the cerebellum	30, -76, 33	174	Deactivated selectively in women for both less-now and more-later decisions in both sessions.
Females only	Main effect of choice	bilateral dorsal anterior cingulate and medial frontal gyrus	16, 36, 36	1222	Deactivation for more-later versus less-now decisions
		bilateral calcarine gyrus and posterior cingulate	2, -63, 22	757	Increased activity for less-now versus more-later decisions
		Right inferior parietal lobe and and angular gyrus	46, -62, 45	399	More deactivation for more-later versus less-now decisions
		Right middle frontal gyrus	44, 40, 26	383	Increased activity for less-now versus more-later decisions
		Left superior and inferior parietal lobe and angular gyrus	-39, -62, 48	225	More deactivation for more-later versus less-now decisions
	Main effect of intervention	Left superior and inferior parietal lobe and angular gyrus	-33, -68, 43	408	Reduced activation during ghrelin versus placebo sessions for both more-later and less-now decisions
		Right superior parietal lobe and superior occipital gyrus	31, -65, 44	288	Reduced activation during ghrelin versus placebo sessions for both more-later and less-now decisions
		Left inferior and middle temporal gyrus	-44, -57, -15	177	Reduced activation during ghrelin versus placebo sessions for both more-later and less-now decisions
Full sample	Main effect of k value during ghrelin session	Left superior parietal lobe and angular gyrus	-49, -64, 42	294	As activity during choice trials increased, k decreased
		Left inferior and middle temporal gyri	-60, -19, 19	447	As activity during choice trials increased, k decreased
	Main effect of k value during placebo session	Left inferior parietal lobe and angular gyrus	-45, -63, 43	199	As activity during choice trials increased, k decreased
		Left middle occipital gyrus	-36, -80, 7	217	As activity during choice trials decreased, so did k values

Table S6. Activation locations for main effects of Choice and Intervention in the Delay Discounting task. All regions surpassed the threshold for whole-brain significance, determined as described in Methods. There was no brain-wide significant region for Choice x Intervention interaction. MNI: Montreal Neurological Institute.

Reported side effects	
**Ghrelin session**	**Saline session**
Muscle twitching during MRI.	Pain in the lower back at the end of the MRI.
Common cold symptoms one day after the session.	Common cold symptoms one week after the session.
Experienced warmth in the beginning of the infusion. Nothing unpleasant.	Sprained toe during training 2 days after the session.
Numbness of the left buttock at the end of MRI.	Left hand went numb during the MRI for about 30 seconds.
Short muscle cramp in the left thigh at the end of MRI.	Muscle twitching during MRI.
Common cold symptoms one day after the session.	Two days after the session noticed redness in left index fingernail.
Dizziness.	Feelings of discomfort during MRI.
Cold sweats.	Headache.
Bright light in the field of vision before MRI, disappeared after ca 30 min.	Increased blood pressure after fMRI. Frightened by the muscle twitches.
Common cold symptoms six days after the session.	Drop in blood pressure during blood draw.
Pressure on the nose from fMRI glasses .	Headache at the end of the session.
Pain from needle insertion site four days after the session.	10 days after the session right hand injury at work.
Nausea a few hours after the session.	

Table S7. Side effects. All reported side effects during and after both the ghrelin and saline session**.**

	**Men**	**Women**	**Total sample**
**Age**	25.3±1.1 [21 – 38]	26.7±3.0 [21 – 55]	26.0±1.4
**BMI**	24.2±0.5 [29.2-19.17]	23.8±08 [29.8 – 18.6]	24.0±0.5

Table S8. Participant characteristics. Values are mean±SEM, with range in brackets.
